# Combination of hepatocyte specific delivery and transformation dependent expression of shRNA inducing transcriptional gene silencing of *c-Myc* promoter in hepatocellular carcinoma cells

**DOI:** 10.1186/1471-2407-14-582

**Published:** 2014-08-10

**Authors:** Mohammad Khalid Zakaria, Imran Khan, Prashant Mani, Parthaprasad Chattopadhyay, Debi P Sarkar, Subrata Sinha

**Affiliations:** Department of Biochemistry, All India Institute of Medical Sciences, New Delhi, 110029 India; Department of Biochemistry, University of Delhi, South Campus, Benito Juarez Road, New Delhi, 110021 India; National Brain Research Centre, Manesar, Gurgaon, Haryana 122050 India

**Keywords:** Hepatocellular carcinoma, Sendai virosome, Asialoglycoprotein receptors, Transcriptional gene silencing, shRNA, *c-Myc*, Alpha-fetoprotein, Heterochromatization, DNA methylation

## Abstract

**Background:**

A specific targeting modality for hepatocellular carcinoma (HCC) could ideally encompass a liver cell specific delivery system of a transcriptional unit that is active only in neoplastic cells. Sendai virosomes, derived from Sendai viral envelopes, home to hepatocytes based on the liver specific expression of asialoglycoprotein receptors (ASGPRs) which are recognized by the Sendai virosomal fusion (F) proteins. As reported earlier by us and other groups, transcriptional gene silencing (TGS) does not require continuous presence of the effector siRNA/shRNA molecule and is heritable, involving epigenetic modifications, leading to long term transcriptional repression. This could be advantageous over conventional gene therapy approaches, since continuous *c-Myc* inactivation is required to suppress hepatocarcinoma cells.

**Methods:**

Exploiting such virosomal delivery, the alpha-fetoprotein (AFP) promoter, in combination with various tumour specific enhancers, was used to drive the expression of shRNA directed against ME1a1 binding site of the proto-oncogene *c-Myc* P2 promoter, in order to induce TGS in neoplastic liver cells.

**Results:**

The dual specificity achieved by the Sendai virosomal delivery system and the promoter/enhancer guided expression ensured that the shRNA inducing TGS was active only in liver cells that had undergone malignant transformation. Our results indicate that such a bimodal therapeutic system induced specific activation of apoptosis in hepatocarcinoma cells due to heterochromatization and increased DNA methylation of the CpG islands around the target loci.

**Conclusions:**

The Sendai virosomal delivery system, combined with AFP promoter/enhancer expression machinery, could serve as a generalized mechanism for the expression of genes deleterious to transformed hepatocarcinoma cells. In this system, the epigenetic suppression of *c-Myc* could have an added advantage for inducing cell death in the targeted cells.

**Electronic supplementary material:**

The online version of this article (doi:10.1186/1471-2407-14-582) contains supplementary material, which is available to authorized users.

## Background

Hepatocellular carcinoma (HCC) is the sixth most prevalent cancer and the third leading cause of worldwide cancer related deaths [1]. Genes of fetal or embryonic origin are often re-expressed in various tumours and alpha-fetoprotein (AFP) expression has been shown to be re-activated in HCC [2]. In cancer gene therapy, the biggest challenges are cell specific targeting and tumour selective expression of the therapeutic gene. A number of reports have raised the issue of specificity and efficiency of gene transfer [3, 4], specifically to neoplastic cells. Specificity can be at two levels. Firstly, it could be at the level of delivery to a particular cell type. Usually, cell type specific antibodies/ligand-receptor units can be used [5–7]. These include binding to generalized ligands like the transferrin or folate receptors [8–11] or antibodies to cell surface antigens [12, 13]. Sendai virosomes specifically fuse with the hepatocytes through their fusion (F) protein’s terminal galactose moiety, that binds specifically with asialoglycoprotein receptors (ASGPRs) present only on the surface of hepatocytes [14]. The Sendai F-virosomal system, lacking the hemagglutinin neuraminidase (HN) protein, is non-toxic and comparatively non-immunogenic. One of us earlier (D.P.S), has successfully demonstrated the expression of human uridinediphosphoglucuronate glucuronosyltransferase-1A1 (hUGT1A1) gene in the hepatocytes of Gunn rats for the treatment of jaundice [15]. The expression of a transgene might be low due to its lysosomal translocation and failure to integrate into the host genome. Sendai F-virosome mediated delivery overcomes this limitation since the entrapped cargo is directly delivered into the cytoplasm, thus evading the endosomal pathway [16, 17]. This could enhance transgene expression and its longevity for therapeutic purposes.

Another level of specificity is at the level of tumour specific promoters [18]. This relies on the fact that several genes, including oncofetal genes are expressed upon cell transformation, implying that the activation of such promoters takes place only in the transformed but not in the normal cells. Such neoplasia activated promoters include carcinogenic embryonic antigen (CEA), prostate specific antigen (PSA), L-plastin, osteocalcin, midkine etc. [19]. For liver neoplasms, it has been shown that an AFP promoter could help achieve a HCC-targeted gene therapy [20–22]. Often, the tumour specific promoters are weak which can be augmented by utilizing various tumour specific enhancers [23] without compromising the specificity. The 5′ flanking region of the AFP gene consists of several enhancer like sequences [24] where one of the core enhancer region can augment gene expression in an engineered construct [25]. However, the possibility of other enhancers could also be explored.

*c-Myc* regulates several cellular processes [26] and is crucial for stem cell maintenance [27]. It is also essential for normal growth and proliferation since its inactivation produces lethal effects [28, 29], indicating its level has to be tightly regulated. Down-regulation of *c-Myc* both *in vitro* and *in vivo* has been shown to induce growth inhibition and differentiation of HCC [30–32]. ME1a1 binding site between P1 and P2 promoter of *c-Myc* is required for sustenance of transcriptionally active dual *c-Myc* promoters [33]. Since the P2 promoter is associated with 75-90% of the *c-Myc* transcripts [26], it serves as an ideal candidate for targeting therapy. We have previously demonstrated that siRNA against *c-Myc* could induce TGS in glioma cells, leading to increased cell death [34].

Post-transcriptional gene silencing (PTGS) involves direct cleavage of the target mRNA by double stranded RNA (dsRNA) [35, 36], whereas Transcriptional Gene Silencing (TGS) induces epigenetic modifications such as CpG methylation and heterochromatization (H3K9Me2 and H3K27Me3) around the target loci [37–40]. The effects of TGS are heritable and lead to long term transcriptional repression of the target gene [41].

In the current study, we have tried to assess the combination of cell type specific delivery and tumour dependent activation for inducing TGS in hepatocellular carcinoma cells. There are no reports of TGS by shRNA driven through a tumour specific promoter delivered by a target specific vehicle. In order to impart strength and specificity to the induction of TGS, we have first generated novel combinations of the AFP promoter with AFP enhancer as well as with the nuclear factor kappa beta (NFκB) response element to drive the expression of shRNA targeting *c-Myc* P2 promoter. Usually shRNA has been expressed by constitutive polymerase (pol) III promoters [42] which fail to elicit tumour specificity. However, in this study, we have tried to achieve specificity as well as efficiency in transcription by using pol II based AFP promoter along with various enhancer elements. Since one of the key events in hepatic oncogenesis is the constitutive activation of NFκB transcription factor [43] and AFP [44], we have compared enhancer systems from both in our study.

Our results indicate that the Sendai virosomal delivery, combined with the AFP promoter/enhancer driven shRNA system, has the necessary specificity and efficiency to activate TGS in hepatocarcinoma cells, leading to cell death. The combination of both targeting entities is likely to be of an added advantage for cancer therapeutics.

## Methods

### Cell culture

HepG2, Huh7, and CHO cells were procured from American type culture collection (ATCC, USA) whereas Chang Liver cells were obtained from National centre for cell sciences (N.C.C.S), Pune. Cells were maintained in Dulbecco’s Modified Eagle’s Medium (DMEM; Sigma-Aldrich, Germany) supplemented with 10% calf fetal serum (Biowest, USA). The molecular characterization of Chang Liver cells was done before any experimentation (Additional file 1: Figure S1).

### Generation of AFP promoter/enhancer +25-luciferase reporter systems

**Figure 1:**
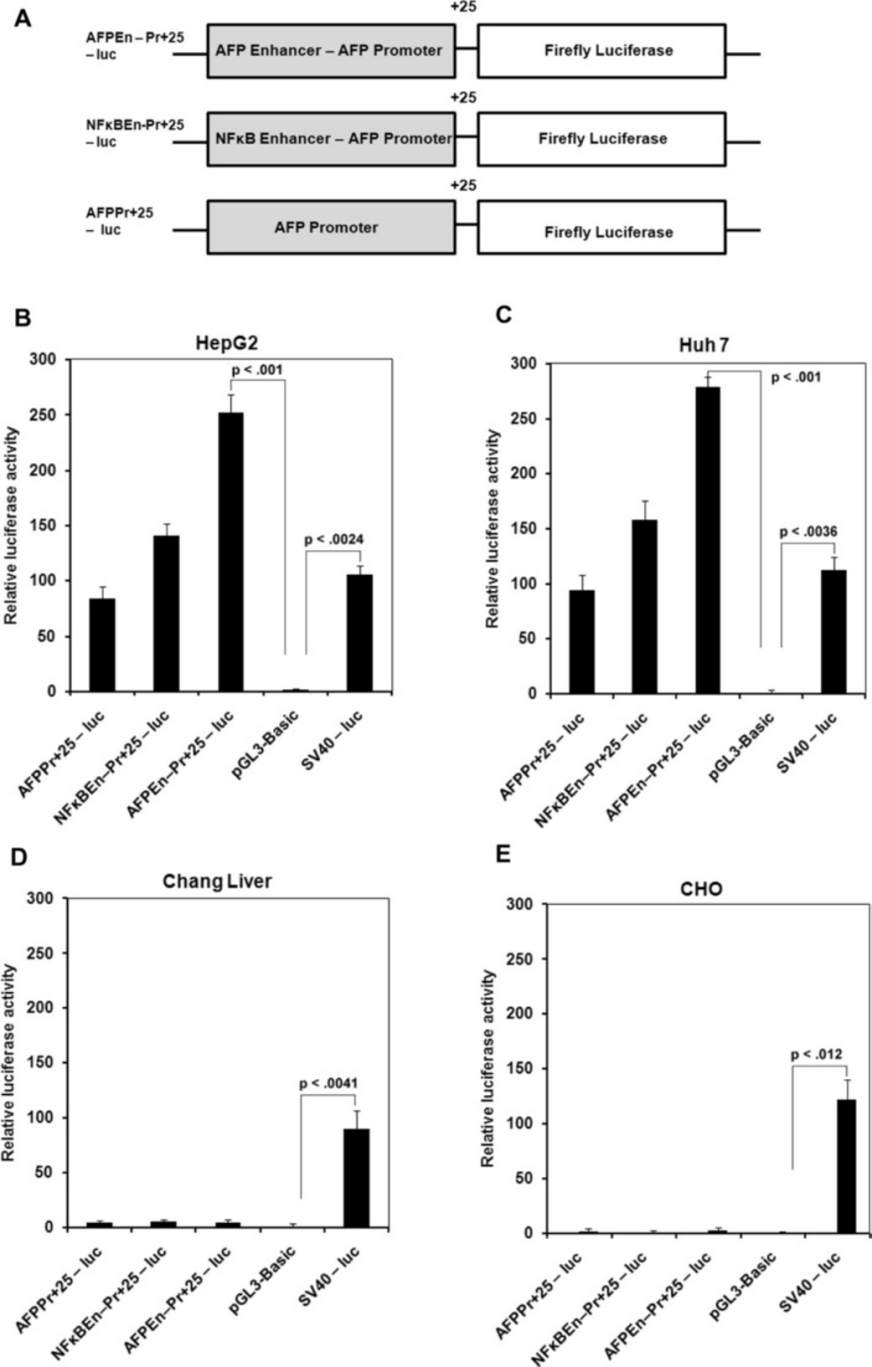
**HCC specific expression of AFP promoter/enhancer system. (A)** Various combinations of AFP promoter/enhancer fusion constructs with downstream luciferase reporter in pGl3-Basic vector. **(B and C)** 48 hours after transfection, the observed luciferase activity was maximum in the case of AFPEn–Pr + 25 – luc followed by NFκBEn–Pr + 25 – luc and lastly by AFPPr + 25 – luc in both HepG2 and Huh7 cells. **(D and E)** No luciferase activity was observed through AFP promoter/enhancer systems in untransformed Chang Liver and non-liver CHO cells.

AFP promoter +25 – luciferase (AFPPr + 25 – luc): AFP promoter region encompassing –230 to +25 base pairs (bp) was PCR amplified using genomic DNA from HepG2 cells with primers having restriction sites MluI and NheI at 5′ and 3′ ends respectively. The PCR amplified product was cloned into pGL3-Basic firefly luciferase reporter vector (Promega, USA) and confirmed both by restriction digestion and DNA sequencing. AFP enhancer – AFP promoter +25 – luciferase (AFPEn-Pr + 25 – luc): Similarly, 700 bp AFP enhancer region was amplified using primers having 5′ KpnI and 3′ MluI restriction sites and cloned upstream to the AFP promoter in pGl3-Basic vector. NFκB enhancer – AFP promoter +25 – luciferase (NFκBEn-Pr + 25 – luc): Four copies of NFκB response elements of ten nucleotides (5′-GGGAATTTCC-3′x 4; [45]) were annealed and cloned upstream to the AFP promoter in pGl3-Basic vector with 5′ KpnI and 3′ MluI sites. Schematic representation of various chimeric AFP promoter driven luciferase reporter constructs are shown in Figure 1A and their clones in Additional file 2: Figure S2. Luciferase reporter under simian virus (SV) 40 promoter (SV40 – luc) served as a positive control.

### Generation of TGS inducing system: AFP promoter/enhancer +2 *c-Myc*shRNA

100 pmoles of both sense and antisense oligonucleotides of *c-Myc* (with pre-added sticky ends; 5′ BamHI and 3′ HindIII) were suspended in 100 μl of 1X Annealing Buffer (10 mM Tris-HCL pH 8.0, 1 mM EDTA and 100 mM NaCl). Oligonucleotides were heated for few minutes in boiling water. Temperature was maintained at 95-100°C for 5 minutes and was allowed to cool over night to room temperature. Agarose Gel Electrophoresis was performed to analyze and excise annealed oligonucleotides.

**Figure 2:**
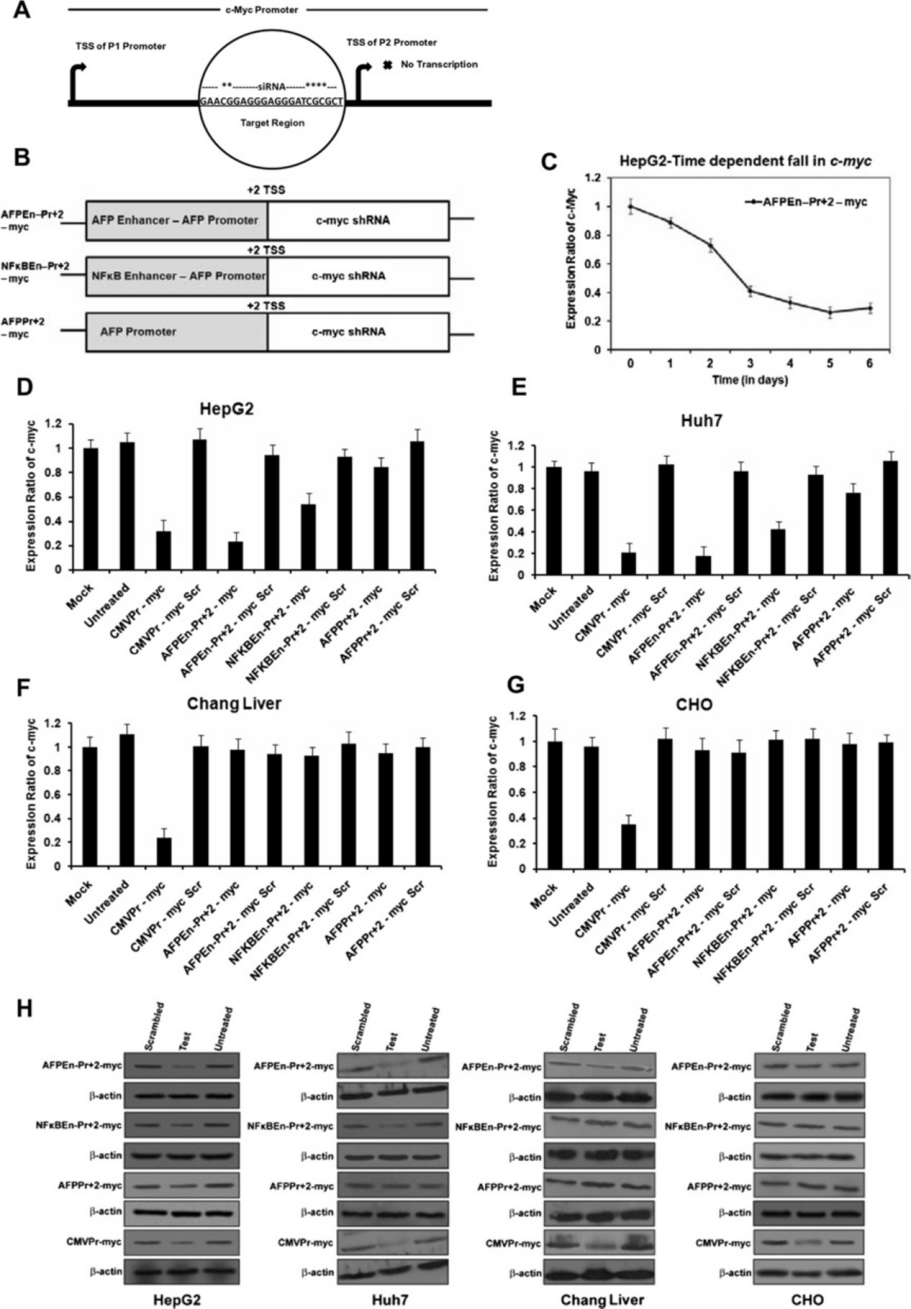
**AFP promoter/enhancer +2 driven shRNA (against** ***c-Myc*** **P2 promoter) decreased** ***c-Myc*** **expression. (A)** siRNA target region on the *c-Myc* P2 promoter. (CpG islands within the target site are marked with *). **(B)** Various AFP promoter/enhancer fusion constructs up to +2 bp relative to the TSS with downstream *c-Myc* shRNA **(C)** Time dependent fall in the expression of *c-Myc,* by AFPEn–Pr + 2 – myc , in HepG2 cells shows maximum suppression after 5 days of shRNA transfection when compared to its scrambled control (p < 0.05 at all-time points). The apparent increase in c-Myc mRNA on the 6^th^ day, when compared with 5^th^ day, was statistically insignificant (p = 0.25). **(D)** Significant decrease in *c-Myc* level was observed in HepG2 by AFPEn–Pr + 2 – myc (p < 0.001) and non- specific positive control CMVPr – myc (p = 0.0026). **(E)** Same trend was observed in Huh7 after 5 days of transfection by the same two constructs (p < 0.001 and p = 0.015). Basal level of *c-Myc*, in Huh7, was lesser when compared to that of HepG2. **(F and G)** No decrease in the expression of *c-Myc* was observed by AFP promoter/enhancer mediated constructs in Chang Liver and CHO cells (p > 0.05 for both) confirming the specificity of the system. However, significant decrease in *c-Myc* level in Chang Liver and CHO cells was observed only through CMVPr – myc (p < 0.001 for both). **(H)** Fall in the expression of c-Myc protein through various shRNA constructs in HepG2, Huh7, Chang Liver and CHO cells corroborated with the RNA level.

Sequence of *c-Myc* promoter region is shown in Additional file 2: Figure S3. shRNA targeting *c-Myc* P2 promoter was designed using Invivogen’s online siRNA wizard (http://www.sirnawizard.com/construct.php) and chemically synthesized by Integrated DNA Technologies, USA. Sequence of the test and scrambled (Scr; control) shRNA is enlisted in Table 1. AFP Promoter, AFP enhancer – AFP promoter and NFκB response element – AFP promoter regions were amplified up to +2 bp relative to the transcription start site (TSS) from the previously generated luciferase reporter constructs (AFPPr + 25 – luc; AFPEn–Pr + 25 – luc; NFκBEn-Pr + 25 – luc respectively) with primers containing 5′ EcoRI and 3′ BamHI restriction sites. Amplification up to +2 bp would minimize sense strand and ensure efficient processing of shRNA by RNAi machinery [46]. These fusion constructs were cloned along with test *c-Myc* shRNA (5′ BamHI and 3′ HindIII sticky overhangs) in shRNA expression vector pSilencer 4.1 (Ambion, USA). The generated constructs are shown in Figure 2B and were as follows: AFP promoter +2 – *c-myc* shRNA (AFPPr + 2 – myc), AFP enhancer – AFP promoter – *c-myc* shRNA (AFPEn–Pr + 2 – myc) and NFκB responsive element – AFP promoter – *c-myc* shRNA (NFκBEn–Pr + 2 – myc). Likewise scrambled *c-Myc* shRNA was cloned downstream to the same promoter/enhancer constructs (AFPPr + 2 – myc Scr; AFPEn–Pr + 2 – myc Scr; NFκBEn–Pr + 2 – myc Scr). *c-Myc* test and scrambled shRNA were also cloned under cytomegalovirus (CMV) promoter (CMVPr – myc and CMVPr – myc Scr respectively) where CMVPr – myc served as a positive control. Annealing of oligonucleotides and schematic representation of all the clones are shown in Additional file 2: Figure S4-S6. All the clones were confirmed by restriction digestion and further authenticated by DNA sequencing from professional agencies.

**Table 1:** **Test and control** ***c-Myc*** **shRNA sequence**

Name	Sequence 5′ to 3′
***c-myc*** **shRNA test sense strand**	**GATCCGAACGGAGGGAGGGATCGCGCTTTTTCAAGAGAAGCGCGATCCCTCCCTCCGTTCTTA**
***c-myc*** **shRNA test antisense strand**	**AGCTTAAGAACGGAGGGAGGGATCGCGCTTCTCTTGAAAAAGCGCGATCCCTCCCTCCGTTCG**
***c-myc*** **shRNA scrambled sense strand**	**GATCCAGCGGTCGAGACGTGGCGGAGATTTTCAAGAGATCTCCGCCACGTCTCGACCGCTTTA**
***c-myc*** **shRNA scrambled antisense strand**	**AGCTTAAGCGGTCGAGACGTGGCGGAGATCTCTTGAAAATCTCCGCCACGTCTCGACCGCTG**

### Transfection

Cells were plated at 10^5^ cells per well in a six-well plate, 3 × 10^5^ cells per 25 cm^2^ flask or 10^6^ cells per 75 cm^2^ flask (Corning, USA). Twenty-four hours later, they were transfected with different reporter or shRNA constructs using Lipofectamine™ 2000 (Invitrogen, USA) as per the manufacturer’s protocol.

### Dual luciferase assay

All the three constructs: AFPPr + 25 – luc, AFPEn–Pr + 25 – luc and NFκBEn–Pr + 25 – luc (Figure 1A) were transfected in HepG2, Huh7, Chang Liver and CHO cells. After 48 hours, transactivation study was done by Dual Luciferase Assay (Promega, USA) following manufacturer’s protocol. The firefly luciferase activity was normalized against Renilla luciferase activity and expressed relative to promoter-less pGl3-Basic control vector. Results are representative of three independent sets of experiments.

### Quantitative RT-PCR to evaluate *c-Myc*down-regulation and shRNA expression

On the 5^th^ and 6^th^ day, post transfection of various test/scrambled shRNA constructs, RNA was isolated from HepG2, Huh7, Chang Liver and CHO cell lines using Trizol (Sigma-Aldrich, Germany). It was treated with DNase (MBI, Fermentas, USA) and quantified by Nanodrop 2000 (Thermo Fischer Scientific, USA). 1 μg of RNA was converted to cDNA using random decamer primers and Moloney Murine Leukemia Virus Reverse Transcriptase (MBI, Fermentas, USA). Real time PCR (RT-PCR) was done on Rotor-Gene 6000 real time PCR machine (Corbett Research, Australia) with the reaction mix containing SYTO 9 green fluorescent dye (Invitrogen, USA). Accurate quantification was done by averaging the geometric mean of multiple internal control reference genes [47] such as β-Actin, 18S, GAPDH and relative expression was estimated by Relative Expression Software Tool (REST; [48]). Primers utilized are given in Additional file 3: Table S1.

Similarly, for *c-Myc* shRNA quantitation, 1st-Strand cDNA Synthesis Kit (Agilent) was utilized, as per the manufacturer’s protocol, and siRNA expression was estimated by RT-PCR. Custom made *c-Myc* siRNA specific primer was obtained separately (sequence in the Additional file 3: Table S1). Luciferase shRNA under CMV promoter (CMV - luc shRNA) served as a control.

### Cell survival assay

2 × 10^4^ cells seeded on to 24 well plates (Corning, USA) were transfected with various AFP promoter/enhancer driven shRNA constructs or their respective scrambled controls. On the 6^th^ day, cells were subjected to MTT (Sigma-Aldrich, Germany) assay for percent cell survival. Furthermore, cell survival was also evaluated by cell counting, post staining with trypan blue (Sigma-Aldrich, Germany), by following manufacturer’s protocol.

### Apoptosis study

10^5^ cells were seeded in 25 cm^2^ cell culture flask (Corning, USA) followed by transfection with various shRNA constructs. On the 6^th^ day, cells were fixed overnight in 70% ice-cold ethanol. Staining of cells was done using Propidium Iodide (PI; Sigma-Aldrich, Germany) and fluorescence was captured using Flow Cytometer (BD Biosciences, USA). Percentage of apoptotic cells (subG1) and other cell cycle phases were estimated using WinMDI software (http://winmdi.software.informer.com/2.8/).

### Western blotting

On the 6^th^ day, post transfection/virosomal delivery of various *c-Myc* shRNA constructs, cell lysates were prepared using triple lysis buffer and protein was estimated by Pierce BCA Protein Assay Kit (Thermo-scientific, USA). Proteins were run on 5% to 12% SDS-PAGE gels and electro transferred to nitrocellulose membranes (Bio-Rad, USA). Blocking was done with 4% bovine serum albumin (Sigma-Aldrich, Germany) and Immunoblotting was done with requisite primary antibodies: anti-actin (sc-8432), anti-*c-Myc* (9E10), anti-TERT (sc-377511) and anti-cyclin D3 (sc-6283); from SantaCruz Biotechnology, USA. Detection of specific proteins was done with horseradish peroxidase (HRP) conjugated secondary antibodies using ECL detection system (Applied Biosystems, USA).

### Sendai virus culture

Sendai virus (Z strain) was grown in 10 - 11 day old embryonated chicken eggs, and extracted by utilizing procedure described in our previous report [16].

### Generation of Sendai fusion (F) virosomes and R18 labeling

Sendai F-virosomes were prepared as described earlier [14]. For Octadecyl Rhodamine B Chloride (R18; Invitrogen, USA) labeling, F-virosome (1 mg/ml) suspension was labeled by adding 10 μl ethanolic soution (1 mg/ml) of R18 in falcon tube while vortex mixing. The mixture was incubated in dark at room temperature for 30 minutes. Excess unbound R18 was removed by ultracentrifugation at 1,00,000 g for 1 hour at 4°C. The pellet was re-suspended in 10 mM phosphate buffered saline (PBS).

### Study of live cell fusion: kinetics of F-virosomes

A measure of Sendai virus fusion with HepG2, Huh7, Chang Liver and CHO cells was done using R18 labeled F-virosomes. Heat inactivation of virosomal F-proteins was performed using procedure described in our earlier reports [16, 17]. HepG2, Huh7, Chang Liver and CHO cells (1 × 10^6^ cells) were incubated with 2 mg of R18 labeled F-virosomes for 1 hour at 4°C. After incubation, cells were centrifuged at 2000 rpm for 5 minutes to remove unbound virosomes. The pellet was suspended in 100 μl of cold 10 mM PBS. For measuring fusion kinetics, 50 μl of the labeled F-virosome-cell complex was added in a cuvette having 3 ml of PBS with 1.5 mM Ca^2+^ (pre-warmed to 37°C). Fusion kinetics was studied by a spectrofluorimeter (FL3-22; Horiba, USA). For data normalization, percent fluorescence dequenching (% FDQ) at a time point was calculated as per the equation: % FDQ = [(F-F_0_)/F_t_ -F_0_)] x 100 where F_0_ denotes fluorescence intensity at time point zero, F is the intensity at a given time point and F_t_ is the intensity recorded when 0.1% Triton X-100 was added to the cell-virosome complex and is designated as fluorescence at “infinite” dilution of the probe (100%).

### Packaging and delivery of AFP promoter/enhancer +2 *c-Myc*shRNA constructs by Sendai F-virosomes

50 mg of Sendai virus envelope was reduced with 3 mM Dithiothreitol (DTT) at 37°C. Viral genetic material and HN were removed from the virosomal suspension by treatment with non-ionic detergent Triton X-100 for 1 hour followed by ultra-centrifugation. From this detergent extract, supernatant was recovered and mixed with required amount of various AFP promoter/enhancer driven *c-Myc* shRNA plasmids. This mixture was reconstituted by step-wise removal of detergent by utilizing SM-2 Biobeads (Bio-Rad, USA). Packaging of shRNA plasmids was confirmed by SDS based lysis and running the contents on 0.8% agarose gel. Cells were plated at 10^5^ cells per well in a six-well plate, 3 × 10^5^ cells per 25 cm^2^ flask, or 10^6^ cells per 75 cm^2^ flask (Corning, USA) followed by transfection with *c-Myc* shRNA loaded F-virosomes.

### CpG methylation study: bisulfite PCR and sequencing

Following virosomal delivery of AFPEn–Pr + 2 – myc or its scrambled control in HepG2 cells, genomic DNA was isolated on the 6^th^ day using Gen Elute Mammalian genomic DNA Miniprep Kit (Sigma-Aldrich, Germany). Bisulfite PCR was done using Epi Tech Bisulfite Kit (Qiagen, Germany) as per the manufacturer’s protocol. http://bisearch.enzim.hu/ was utilized for designing specific primers. Primers were M13-tagged for sequencing of PCR products.

### Assessment of heterochromatization by chromatin immunoprecipitation (ChIP) assay

Chromatin immunoprecipitation (ChIP) assay for H3K9Me2 and H3K27Me3 was done using EZ ChIP kit (Millipore, USA) as per manufacturer’s protocol. Input DNA, anti-H3K9Me2 (mAbcam1220), anti-H3K27Me3 (mAbcam6002), anti-histone 3 acetylated (Upstate) and control mouse IgG antibody (Upstate) immunoprecipitated DNA was amplified using primers specific for the target region on the *c-Myc* P2 promoter listed in Additional file 3: Table S1. Immunoprecipitation percentage was calculated as described by Haring et al. [49]. Centrosome of chromosome 16 served as a positive control, since it has 100% methylated histone tails.

### Suppression of histone deacetylase (HDAC) and DNA methyl transferase (DNMT)

Trichostatin A (TSA; Sigma-Aldrich, Germany; 300nM) and 5-aza-2-deoxycytidine (AZA; Sigma-Aldrich, Germany; 5 mM) were prepared as per manufacturer’s datasheet. Cells were pre-treated with TSA/AZA or both for 48 hours followed by virosomal delivery of the AFPEn–Pr + 2 – myc or its scrambled control.

### Caspase 3/7 assay for evaluation of apoptosis after virosomal delivery of shRNA

Caspase 3/7 activity of HepG2, Huh7 and Chang Liver cell lines was measured post virosomal delivery of AFPEn–Pr + 2 – myc or AFPEn–Pr + 2 – myc Scr by using caspase 3/7 assay kit (Promega, USA) as per manufacturer’s protocol.

### Statistical analysis

All experiments including dual luciferase assay, cell survival assays and RT-PCR was repeated thrice and performed in triplicates. Western blotting, virosome fluorescence dequenching assay, Flow cytometric analysis, Bisulfite PCR, ChIP assay and capase 3/7 assay were repeated at least twice. Student’s t-test was utilized to calculate the significance in all experiments and p < 0.05 was considered significant whereas p < 0.001 as highly significant. The data are shown as mean ± SD.

## Results

### Characterization of the novel NFκB/AFP enhancer – AFP promoter +25 based constructs

The AFP enhancer – AFP promoter +25 (AFPEn-Pr + 25), NFκB response element – AFP promoter +25 (NFκBEn-Pr + 25) and AFP promoter +25 (AFPPr + 25) generated constructs (Figure 1A) were verified by sequencing. The sequence encompassing different restriction sites on pGl3-Basic vector are given in Additional file 4: Figure S7.

### AFP promoter/enhancer mediated expression is hepatocarcinoma specific

The generated luciferase constructs were transfected in both transformed and untransformed cell lines and their proficiency was determined by dual luciferase assay after 48 hours. In the transformed HCC cells, HepG2 and Huh7, the luciferase activity was highest with AFPEn–Pr + 25 – luc followed by NFκBEn–Pr + 25 – luc and lastly by AFPPr + 25 – luc, indicating the relative activity of the AFPEn–Pr + 25 in the transformed cells is significantly higher than SV40 promoter (Figure 1B and C). However, in the untransformed Chang Liver and non-hepatic CHO cells, significant activity was observed only with SV40 – luc and not in case of AFP promoter/enhancer constructs (Figure 1D and E).

### Decrease in *c-Myc*level by TGS inducing shRNA

Various *c-Myc* shRNA constructs, against *c-Myc* P2 promoter (Figure 2A), were generated as described in methods (Figure 2B). AFPEn–Pr + 2 – myc and AFPEn–Pr + 2 – myc Scr were transfected in HepG2 cells and fall in the expression of *c-Myc* was evaluated consecutively for 6 days by RT-PCR (Figure 2C). The decrease in c-Myc mRNA level was significant at all-time points (p < 0.05) with respect to its control and was maximum on the 5^th^ day. Slight apparent increase on the 6^th^ day when compared to that of 5^th^ day was insignificant (p = 0.25). Similarly, fall in the *c-Myc* expression, by other shRNA constructs was also evaluated 5 days post transfection in HepG2 cells (Figure 2D). Similar results were observed for Huh7 cells (Figure 2E). However, the absolute levels of *c-Myc* were higher in HepG2 as compared to Huh7. No significant decrease in *c-Myc* was observed in the Chang Liver and CHO cells (p > 0.05 for both; Figure 2F and G). The levels of c-Myc protein (Figure 2H) corroborated with mRNA data but the tissue non-specific CMV promoter driven *c-Myc* shRNA (CMVPr – myc) decreased the level of *c-Myc* even in Chang Liver and CHO cells (p < 0.001 for both; Figure 2F and G).

### TGS of *c-Myc*reduced cell survival and increased apoptosis

**Figure 3:**
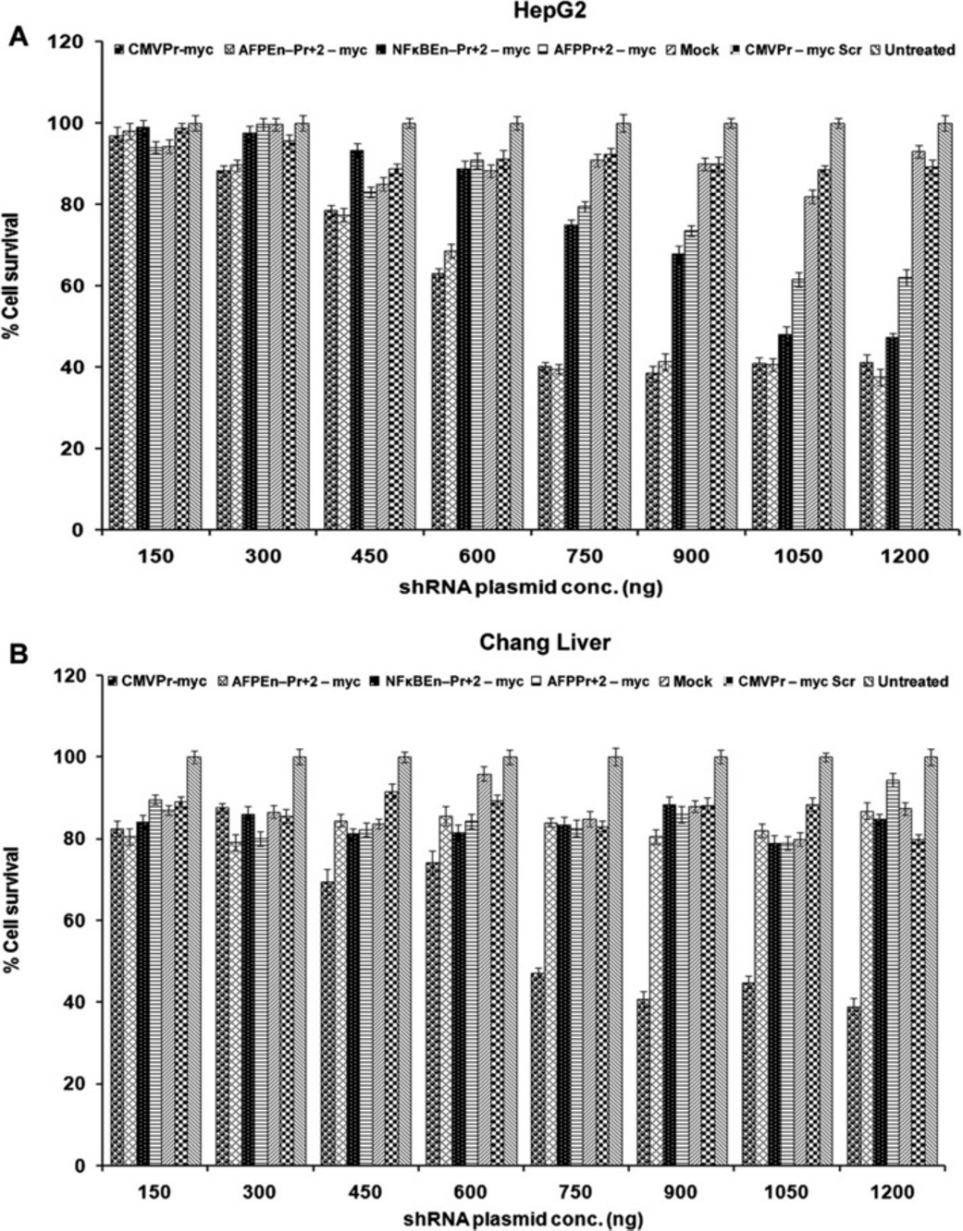
***c-Myc*** **suppression by TGS reduced cell survival. (A)** HepG2 cells were transfected with various AFP promoter/enhancer driven test/control shRNAs in different doses and percent cell survival was evaluated by MTT assay on the 6^th^ day. HepG2 cells showed decrease in cell survival with the increasing dose of the constructs and this decrease in cell survival was dependent upon the strength of each construct when compared to its control (p < 0.05). **(B)** In untransformed Chang Liver cell line, decrease in cell survival was observed only by CMVPr – myc (p = 0.019).

**Figure 4:**
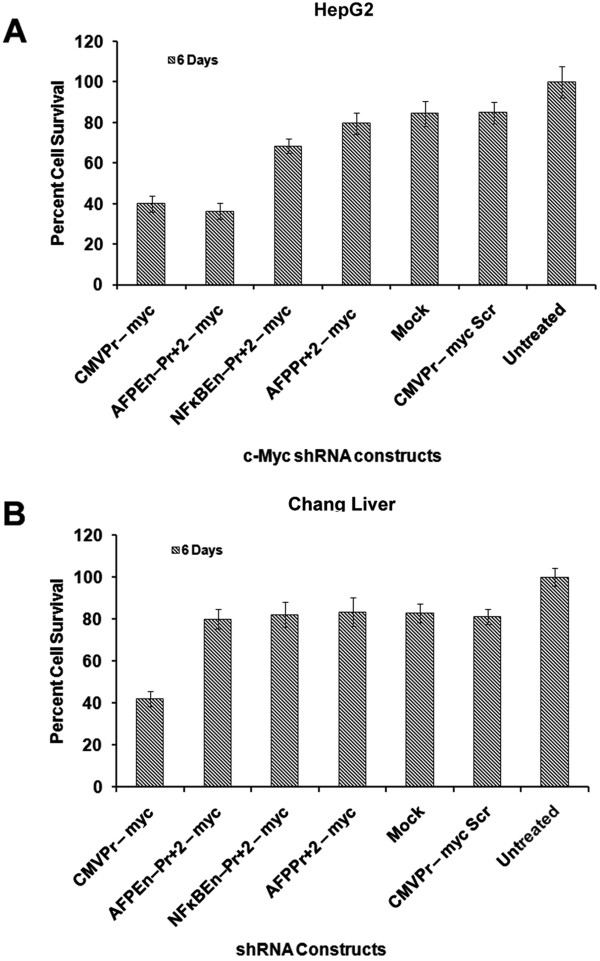
**Estimation of cell survival by trypan blue staining. (A)** Trypan blue cell counting assay was done, on the 6^th^day, post transfection of all AFP promoter/enhancer driven *c-Myc* shRNA constructs in HepG2 cells. The decrease in cell survival corroborated with the MTT assay and was found to be significant, when compared to their respective scrambled controls (p < 0.05). **(B)** In the case of Chang Liver cells, significant decrease in the cell viability was observed only by CMVPr – myc (p < 0.001) as the AFP promoter enhancer system was inactive in it.

To examine whether decrease in the expression of *c-Myc,* by TGS, affects cell growth, both cell survival and apoptosis were evaluated. MTT assay, on the 6^th^ day post shRNA transfection, revealed decrease in cell survival of the transformed cell line HepG2 and Huh7 (p < 0.05 for both; Figure 3A and Additional file 4: Figure S8), however, Huh7 cells were less responsive to *c-Myc* knockdown. No such inhibitory effects were observed in the case of untransformed Chang Liver cell line. On the other hand, CMVPr – myc showed significant cell killing and suppression even in Chang Liver cells due to its non-specific nature (p = 0.019; Figure 3B). Cell survival, of HepG2, Huh7 and Chang Liver cells, was further estimated by trypan blue staining followed by cell counting, which corroborated with the MTT data (Figure 4 and Additional file 4: Figure S9; p < 0.05). Any molecular analysis beyond 6 days was not possible due to complete detachment of HepG2 cells treated with the AFPEn–Pr + 2 – myc when compared to the scrambled control.

**Figure 5:**
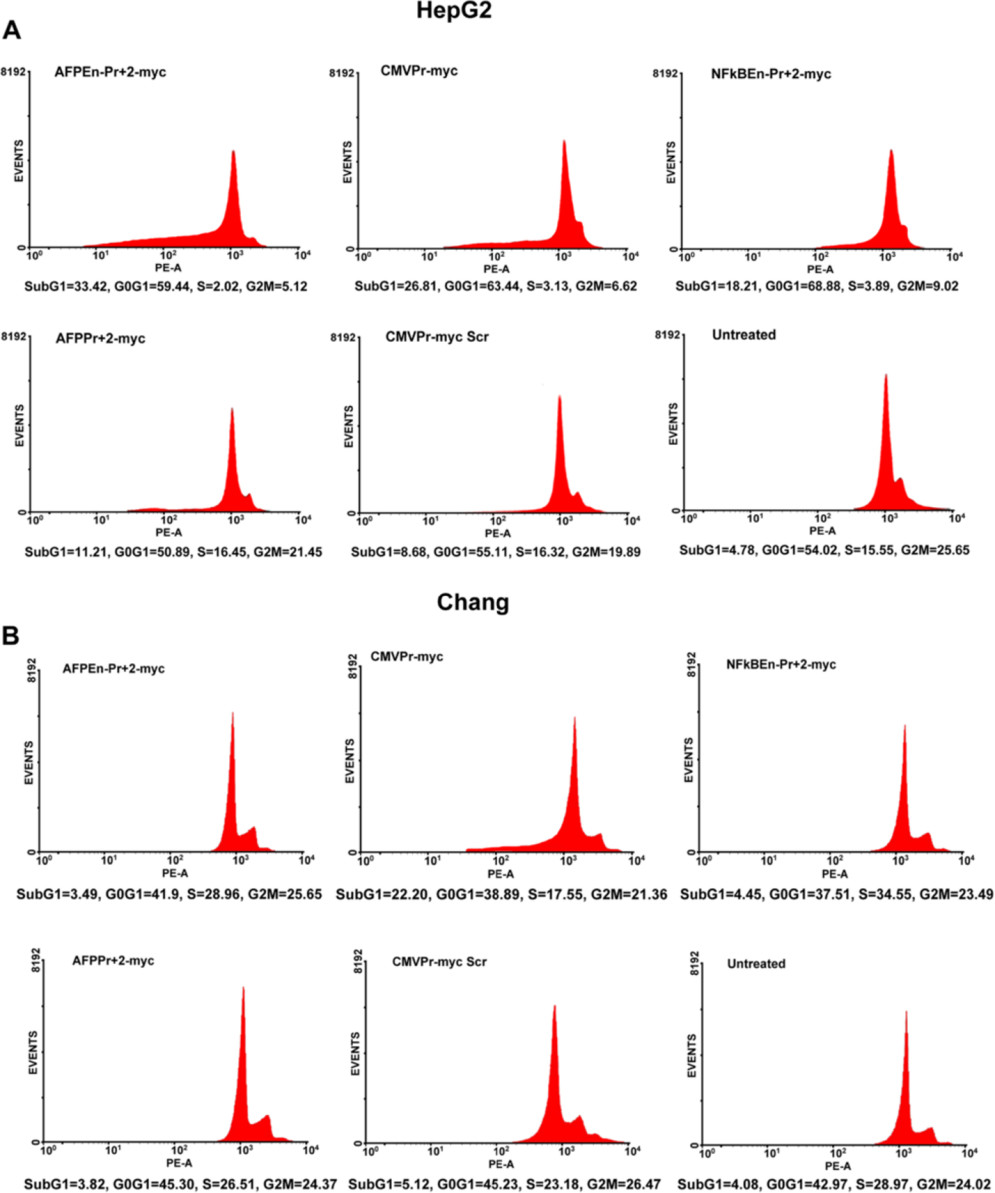
**Increased apoptosis upon TGS of** ***c-Myc*** **. (A)** *c-Myc* shRNA constructs were transfected in HepG2 cells and post 5 days, apoptosis (subG1) was evaluated by flow cytometry. Increase in apoptosis was found to be concordant with the strength of promoter/enhancer based construct driving shRNA transcription and corroborated with the MTT data. Since the fixed amount of each construct was utilized, maximum percentage of cells in subG1 phase was observed through AFPEn–Pr + 2 – myc construct whereas it was the lowest in the case of AFPPr + 2 – myc. No significant apoptosis was seen by the scrambled *c-Myc* shRNA under CMV promoter (CMVPr – myc Scr). In addition, *c-Myc* suppressed cells showed reduced S and G2M phase. **(B)** In the case of Chang Liver cell line, only CMVPr – myc clearly shows significant population of apoptotic cells due to its non-specific nature.

Flow cytometric studies by PI staining showed that the percentage of apoptotic cells (sub G1 proportion) in HepG2 was in concordance with the strength of the AFP promoter/enhancer constructs driving the shRNA expression (Figure 5A). Similar trend was observed in the case of Huh7 cells but to a lesser degree (Additional file 4: Figure S10). Significant apoptosis in Chang Liver was seen only by CMVPr – myc and not by any of the AFP promoter/enhancer mediated *c-Myc* shRNA constructs (Figure 5B). *c-Myc* suppressed cells (HepG2 and Huh7), in addition to apoptosis (subG1 proportion), were found to be within the G0-G1 phase with decreased S and G2M phase. Suppression of *c-Myc*, by TGS, had a profound effect on the cell survival and apoptosis of HepG2 cells when compared with that of Huh7.

### Specific binding of Sendai F-virosomes to cells of liver origin

**Figure 6:**
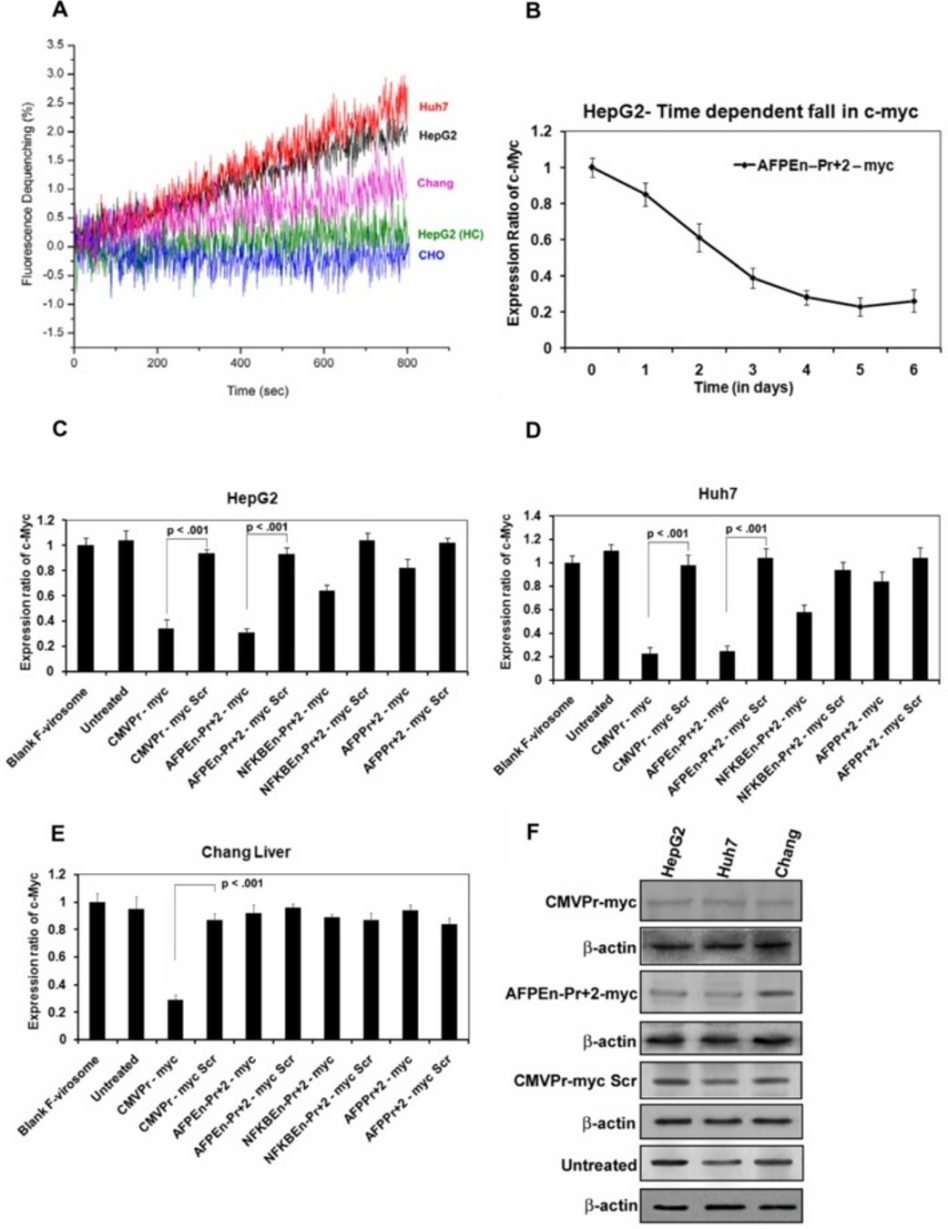
**AFP promoter/enhancer +2** ***c-My c*** **shRNA delivered by F-virosomes down-regulated** ***c-Myc*** **. (A)** Hemi fusion study in various hepatoma, untransformed and non-liver cells was done by fluorescence dequenching assay. Fusion of R18 labeled Sendai F-virosomes was determined by spectro-fluorimetry and was almost similar in the case of HepG2 and Huh7, whereas it was slightly lesser with Chang Liver cells. CHO cells, being a non-liver cell line, lack ASGPR and served as a negative control. F-virosomes with inactivated F-protein (HC: Heat Control) displayed poor fusion with the HepG2 cells. **(B)** Time dependant fall in the expression of *c-Myc* by AFPEn–Pr + 2 – myc after virosomal delivery to HepG2 cells was significantly comparable with that of Lipofectamine^TM 2000^ **(C)** In HepG2 cells, AFPEn–Pr + 2 – myc construct decreases *c-Myc* level significantly which was comparable to that of the positive control CMVPr – myc. **(D)** Similar pattern was observed in the case of Huh7. **(E)** Down-regulation of *c-Myc* in untransformed Chang Liver cell line was observed only by CMVPr – myc and not by AFP promoter/enhancer driven shRNA. **(F)** Western Blot Analysis of *c-Myc* in HepG2, Huh7 and Chang Liver was in concordance with real-time PCR analysis and followed the same trend.

Once the specificity of *c-Myc* suppression in HCC cell lines was established, we aimed to increase the level of specificity further by packaging the AFP promoter/enhancer shRNA constructs within the Sendai virosomes for liver specific delivery. Real time fusion kinetics by fluorescence dequenching assay revealed that Sendai F-virosomes bind specifically to hepatic cells (HepG2, Huh7 and Chang Liver) and not with control non hepatic cell line CHO. Virosomes with inactivated F-proteins (HC: Heat control), displayed poor fusion even with HepG2 cells, confirming the specific fusion *via* F-protein and ASGPR of the hepatocytes (Figure 6A). The difference in the fusion observed might be dependent upon the number of ASGPRs expressed by various cell types.

Once significant fusion was confirmed, the generated constructs were packaged and delivered by Sendai F-virosomes to both transformed and untransformed liver cells. Time dependent fall in the *c-Myc* level post virosomal delivery in HepG2 cells (Figure 6B) was highly comparable to that by conventional method (Figure 2B). Maximum suppression of *c-Myc* was observed on the 5^th^ day with AFPEn–Pr + 2 – myc and slight increase on the 6^th^ day when compared to the 5^th^ was insignificant (p = 0.41). Significant fall in the expression of c-Myc mRNA was seen both in HepG2 and Huh7 by other AFP promoter/enhancer constructs, (p < 0.05 for both; Figure 6C and D). Even though the fluorescence dequenching experiments demonstrated fusion of F-virosomes with Chang Liver, TGS was not effective in these cells due to inactivation of AFP promoter/enhancer system (Figure 6E). Decrease in c-Myc protein levels were in concordance with its mRNA levels (Figure 6F).

### No interferon response is mounted by *c-Myc*shRNA

**Figure 7:**
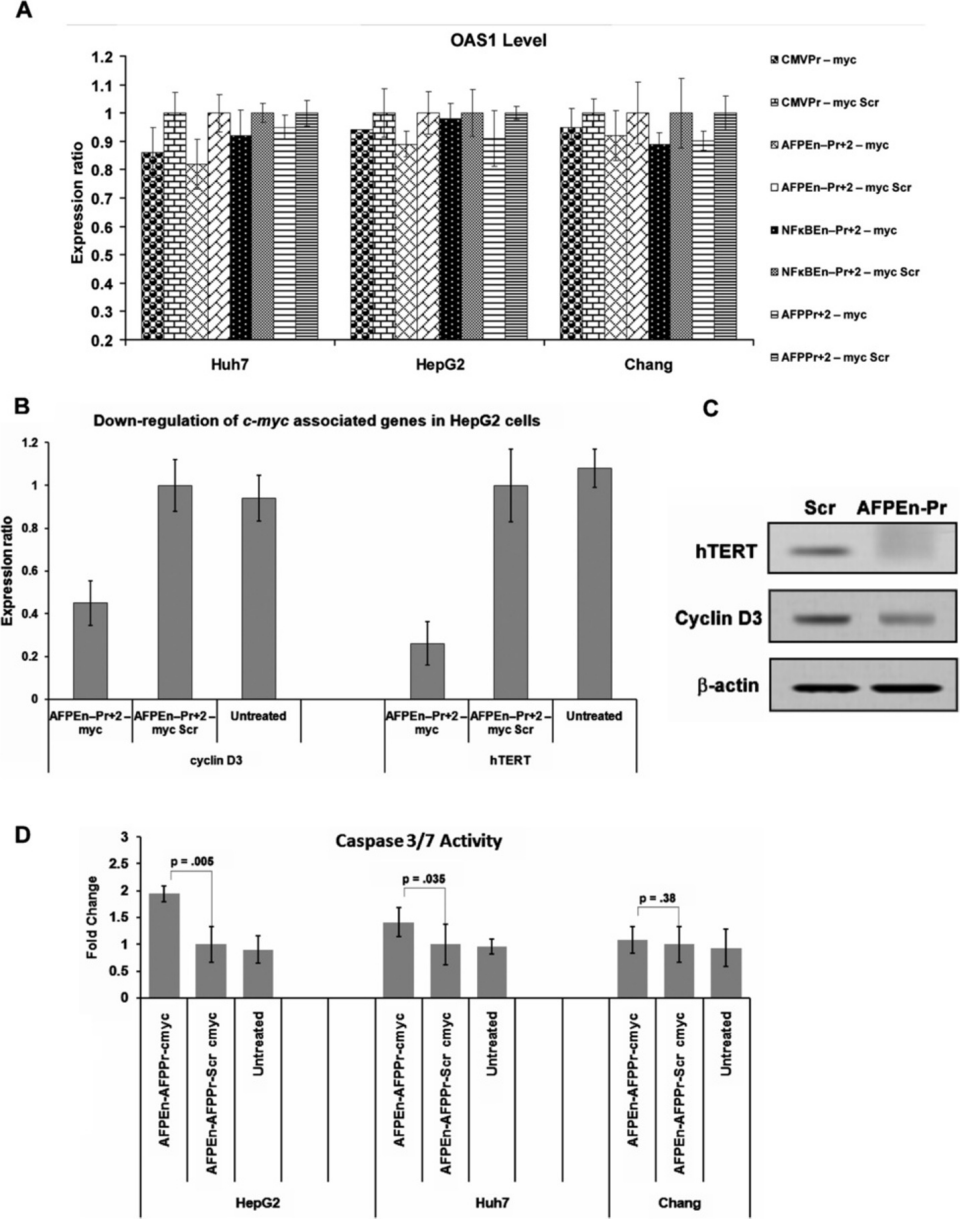
**Evaluation of interferon response, Cyclin D3, hTERT and caspase activity post TGS of** ***c-Myc.*** **(A)** No Significant induction of OAS1 levels were seen in HepG2, Huh7 and Chang Liver cells, post 5 days of virosomal delivery, by chimeric AFP promoter driven shRNA constructs, confirming the absence of interferon response (p > 0.05 at all point). **(B)** Decrease in the RNA level of both Cyclin D3 and hTERT (p = 0.0022 and p < 0.001 respectively) following *c-myc* suppression was seen in HepG2 cells through AFPEn–Pr + 2 – myc when compared to its control AFPEn–Pr + 2 – myc Scr. **(C)** Similar results were observed at the protein level. **(D)** *c-Myc* suppression by AFPEn–Pr + 2 – myc, post 5 days after virosomal delivery, led to increase in caspase 3/7 activity in HepG2 cells (p = 0.005) and Huh7 cells (p = 0.035). However, increase in caspase 3/7 activity in Huh7 was to a lesser degree. No such increase was observed in the case of Chang Liver cell line (p = 0.38).

Entry of dsRNA into the cell might lead to non-specific interferon (IFN) responses [50] which involves the activation of the PKR/RNase L pathway ultimately inducing an IFN marker 2,5-oligoadenylate synthetase 1 (OAS1) [51]. There was no significant induction of OAS1 in HepG2, Huh7 and Chang Liver cells (p > 0.05 at all points) post 5 days of shRNA delivery through F-virosomes; indicating the absence of an IFN response (Figure 7A). Furthermore, no significant increase in the levels of OAS1 was observed in 24, 48, 72 and 96 hours (p > 0.05 at all points) after similar treatment of HepG2 cells (Additional file 4: Figure S11), ruling out IFN response being generated even at earlier time points following F-virosomal delivery of the entrapped shRNA constructs.

### *c-Myc*inactivation caused down-regulation of other proliferative genes

*c-Myc* regulates growth and proliferation by regulating various genes [26]. Cyclin D3 as well as human telomerase reverse transcriptase (hTERT) were studied in HepG2 cells at both mRNA and protein level. Fall in *c-Myc* by F-virosomes loaded with AFPEn–Pr + 2 – myc led to significant decrease in Cyclin D3 and hTERT both at mRNA (p = 0.0022 and p < 0.001) and protein levels, suggesting the down-regulation of *c-Myc* effector molecules (Figure 7B and C).

### Increase in caspase 3/7 activity following TGS of *c-Myc*

To validate the activation of apoptosis after *c-Myc* suppression by chimeric AFP promoter driven *c-Myc* shRNA, caspase 3/7 activity was evaluated in HepG2, Huh7 and Chang Liver cell lines, 5 days after virosomal delivery of AFPEn–Pr + 2 – myc (Figure 7D). The increase in caspase activity was in agreement with the magnitude of chimeric AFP promoter driving the shRNA. In HepG2 cells, significant increase in caspase 3/7 activity was observed (p = 0.005) as compared to its scrambled control, however, activation of caspase 3/7 was to a lesser degree in Huh7 (p = 0.035). No increase in the activity was seen in Chang Liver cells (p = 0.38).

### shRNA induced TGS by chromatin condensation and CpG methylation of *c-Myc*P2 promoter

**Figure 8:**
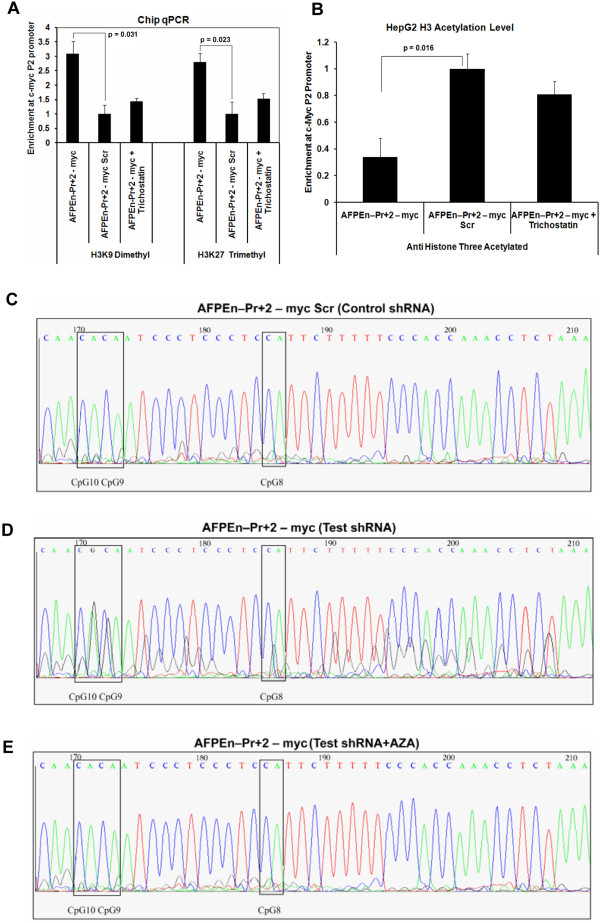
***c-Myc*** **shRNA induced epigenetic modifications around the target loci. (A)** As evaluated by Chip Assay followed by qPCR, significant enrichment of both H3K9Me2 (p = 0.031) and H3K27Me3 (p = 0.023) was found at *c-Myc* P2 promoter on the 6^th^ day after virosomal delivery of AFPEn–Pr + 2 – myc, whereas its scrambled control did not elicit the same level of enrichment. However, HepG2 cells pre-treated with TSA, did not show significant enrichment of both H3K9Me2 (p = 0.55) and H3K27Me3 (p = 0.37) by AFPEn–Pr + 2 – myc shRNA construct. This indicates that in the presence of TSA, shRNA failed to induce significant heterochromatization around the target site. **(B)** 6 days post transfection of AFPEn–Pr + 2 – myc in HepG2 cells, acetylation status of the *c-Myc* P2 promoter was evaluated by utilizing anti-histone 3 acetylated antibodies for ChIP assay followed by quantitative RT-PCR. The acetylation level significantly reduced post shRNA treatment (p = 0.016). However, no decrease in the acetylation level, by shRNA, was observed in the TSA treated HepG2 cells when compared to the scrambled control (p > 0.05). This indicates shRNA mediated possible recruitment of HDACs at the target site causing de-acetylation, which was reversed upon treatment with TSA. **(C, D and E)** On the 6^th^ day following F-virosomal delivery of AFPEn–Pr + 2 – myc in HepG2 cells, bisulfite PCR products were analyzed for methylation by DNA sequencing. **(C)** Sequence chromatogram result shows that methylation was induced by test *c-Myc* shRNA on CpG 8, 9 and 10 of *c-Myc* P2 promoter. **(D)** No methylation was induced by control shRNA. **(E)** Cells pretreated with AZA shows no methylation even by the test shRNA, indicating failure in the recruitment of DNMTs by the shRNA at the target site.

To evaluate the mechanism by which shRNA acted on the target region, the chromatin status of the *c-Myc* P2 promoter was evaluated by ChIP assay, on the 6^th^ day, post virosomal delivery of the AFPEn–Pr + 2 – myc construct in HepG2 cells. ChIP followed by quantitative RT-PCR revealed that *c-Myc* shRNA mediated TGS was associated with H3K9 dimethylation and H3K27 trimethylation. Cells pre-treated with HDAC inhibitor TSA showed reduced enrichment of histone chromatin marks even in the presence of AFPEn–Pr + 2 – myc. This indicated the likely involvement of HDACs in gene silencing of *c-Myc* (Figure 8A). Similarly, we checked the acetylation status of the target region following AFPEn–Pr + 2 – myc transfection, by utilizing anti-histone 3 acetylated antibodies. Significant decrease in the acetylation level was observed post *c-Myc* suppression on day 6 (Figure 8B; p = 0.016). However, in the presence of TSA, no decrease was observed (p > 0.05) as the shRNA failed to recruit HDACs.

Furthermore, the methylation status of CpG islands was checked by bisulfite PCR followed by DNA sequencing. Methylation of CpG 8, 9 and 10, when compared to scrambled control, was observed in the test shRNA treated cells (Figure 8C and D). Moreover, such effect was abrogated by pre-treatment of HepG2 cells with DNMT inhibitor AZA, confirming the possible recruitment of DNMTs, by shRNA, to the target site (Figure 8E).

We also determined the effect of TSA/AZA or both in combination on *c-Myc* transcription in HepG2 cells by RT-PCR. Cells pre-treated with both AZA and TSA showed no significant decrease in *c-Myc* levels by AFPEn–Pr + 2 – myc on the 6^th^ day after treatment. Additionally, when the cells were pretreated with AZA or TSA individually, AFPEn–Pr + 2 – myc down-regulated *c-Myc* levels significantly, indicating that both HDACs and DNMTs are involved in gene silencing of *c-Myc* (Additional file 5: Figure S12).

It is known that TGS can continue for a significant number of days after transfection [39, 52, 53]. In this study, we performed real time PCR to study the dynamics of *c-Myc* mRNA as well as shRNA expression after transient transfection of various shRNA constructs in HepG2 cells. For AFPEn–Pr + 2 – myc construct, shRNA was maximally expressed after 48 hours while declining to around 18% of the maximum on day 6 (Additional file 5: Figure S13). On day 6, c-Myc mRNA was continued to be suppressed (Figure 2C and 6B) and all the molecular markers of TGS were present (Figure 8). On day 7, almost all the cells detached from the culture plate due to extensive cell death, making it impossible to do any mRNA/shRNA quantitation. This supports the possibility that TGS continues even after the reduction of shRNA, even though because of cell death, we were unable to reach zero expression.

## Discussion

Specificity is the cornerstone of cancer therapy and a considerable part of the current research on cancer therapeutics tries to address this issue in the context of efficacy. In this study, we have tried to combine modalities for achieving specificity at two levels – that of the delivery system as well as the transcription of its cargo. This approach has been utilized for the expression of shRNA for inducing the suppression of *c-Myc* by TGS. Although majority of the *c-Myc* transcripts are P2 promoter driven [26], targeting approaches are hindered by the lack of specificity. Since *c-Myc* is required for normal growth and proliferation, its non specific suppression might lead to hazarduous effects [54].

Sendai virosomes are naturally hepatotropic in nature because of their internalization through the ASGPRs of hepatocytes [14]. One of us has earlier described their properties both *in vitro* and *in vivo* and has used this system for gene delivery to hepatocytes in the Gunn rat model with good efficacy [15]. Sendai virosomes were shown to have high degree of direct cytoplasmic delivery with low immunogenicity [15–17].

At the second level of specificity we have tried to use liver tumour specific AFP promoter based fusion constructs. The AFP promoter has been used earlier to drive specific genes, mostly apoptotic or pro-drug metabolizing enzymes in hepatoma cells [55–59]. However, in our study, we have taken the minimal AFP promoter and added upstream enhancer regions from the AFP gene itself and, in another construct, the NFκB response element. This was done to increase the extent of promoter specific gene expression. Our studies showed that the AFP promoter fused with AFP enhancer (AFPEn–Pr + 25), had the strongest and specific expression in HCC cells.

As demonstrated by Dual Luciferase Assay, various AFP promoter based enhancer sytems specifically and optimally expressed luciferase in hepatoma models HepG2 and Huh7 but not in untransformed Chang Liver and non liver CHO cells (Figure 1B-E). Only the positive control construct (SV40 – luc) expressed luciferase in both Chang Liver and CHO cells because of its nonspecific nature (Figure 1D and E).

The specially designed AFP promoter/enhancer driven *c-Myc* shRNA encompassing ME1a1 site upstream of *c-Myc* P2 promoter resulted in reduced *c-Myc* expression only in transformed hepatocarcinoma cells (Figure 2D and E). However, due to its universal nature, CMVPr – myc decreased the level of *c-Myc* even in Chang Liver and CHO cells (Figure 2F and G). The suppression of *c-Myc* in transformed cells was in concordance with the strength of each construct (Figure 2D-G). The AFP Enhancer – AFP promoter construct was equivalent in strength to the known constitutive viral promoter CMV and stronger than SV40, while retaining specificity for HCC cells. However, Huh7 having lower basal level of *c-Myc* compared to HepG2 was less responsive to *c-Myc* suppression. Previous studies have shown that *c-Myc* could abrogate the p53-induced cell-cycle arrest [60], and it is possible that HepG2 cells, which contain wild-type p53 compared to mutant p53 in Huh7 [61], were more sensitive to *c-Myc* suppression. Additionally, increased activity of Wnt/β-catenin pathway in HepG2 than Huh7, which is a direct regulator of *c-Myc* [62], also might add on to the greater *c-Myc* level and its consequent implications in HepG2.

*c-Myc* suppressed cells showed decreased cell survival and increased apoptosis, as evaluated by MTT Assay and Flow Cytometric analysis respectively (Figures 3 and 5). Moreover, cell survival estimated by trypan blue cell counting corroborated with the MTT data (Figure 4). This was concordant with the strength of promoter/enhancer construct driving shRNA expression. The effect on HepG2 cells (Figures 3A, 4A and 5A) were more pronounced than that of Huh7 cells (Additional file 4: Figure S8, S9 and S10). However, no decrease in cell viability was observed in the case of Chang Liver cells as the AFP promoter based system was inactive in these cells (Figures 3B and 4B). Moreover, the specificity for transformed hepatocytes was clear as *c-Myc* shRNA under the CMV promoter induced apoptosis even in Chang Liver cells (Figure 5B). Due to *c-Myc* suppression, via TGS, majority of the transformed cells were found to be present within the subG1 phase followed by G0-G1 phase.

Since the use of antisense oligonucleotides or siRNA/shRNA is potentially limited by ineffective delivery into cancer cells [63], to ensure specific and substantial level of therapeutic entry, shRNA constructs were packaged and delivered to various cell lines through Sendai F-virosomal system. Post virosomal delivery, the reduction in the level of *c-Myc* was significantly comparable to that by conventional transfection reagent (Figures 2 and 6).

*c-Myc* shRNA did not induce IFN response since there was no significant increase in the level of IFN marker OAS1 in HepG2, Huh7 and Chang Liver cells, post 5 days of virosomal delivery (Figure 7A) as well as at earlier time points (up to 4 days; Additional file 4: Figure S11 ). Some of the *c-Myc* effector molecules are hTERT [64] and Cyclin D3 [65]. Although hTERT is not oncogenic per se, the activation of hTERT is essential for maintaining neoplastic transformation [66]. Following virosomal delivery of AFPEn–Pr + 2 – myc, significant decrease in hTERT and Cyclin D3 mRNA and protein was observed in HepG2 cells following *c-Myc* suppression (Figure 7B and C). Furthermore, the more pronounced increase in caspase 3/7 activity in HepG2 and not in Chang Liver was in agreement with Flow cytometric studies (Figure 7D).

Earlier reports of TGS have shown that silencing occurs through histone modifications [67–69], CpG methylation [70, 71] or interference of RNA polymerase binding [52]. In our case, we could demonstrate the induction of TGS by both heterochromatization and DNA methylation. Previously, other groups have targeted different regions of *c-Myc* promoter by siRNAs. siRNA against *c-Myc* transcription start site has shown promising results in suppressing prostate cancer cells, for a longer duration, by interfering with the binding of RNA polymerase [52]. The same group has recently shown an effective strategy in suppressing prostate cancer stem cells, with good efficacy, both in culture and in mouse model through the promoter directed siRNAs [72]. Small molecule inhibitor of *c-Myc* has proved useful in suppressing as well as chemo sensitizing HepG2 cells towards conventional drugs [73]. Additionally, several reports have demonstrated that suppression in *c-Myc* levels induces shrinkage in tumour volume [30–32].

In published literature, it is indicated that while PTGS would require sustained presence of the effector siRNA molecule, TGS would be long lasting, by virtue of its capability to induce heritable epigenetic changes [37, 74]. Hence PTGS would also work in this cell specific promoter/delivery system albeit possibly for a shorter duration. However, we have not demonstrated the same experimentally. There is a report that after 7 days of continuous induction by siRNA against human ubiquitin c gene’s (UbC) promoter, TGS persisted for over a month [39]. In a recent study from our lab, TGS of HIV clade C LTR was shown to be effective for at least 21 days after siRNA transfection [53].

In our study, we could follow the expression of *c-Myc* mRNA and shRNA for only 6 days after transfection (Figures 2C and 6B and Additional file 5: Figure S13). Extensive cell death, of HepG2 cells, prevented us from quantifying mRNA and shRNA levels on day 7 and beyond. While on day 6, shRNA levels were around 18% of the maximum (on day 2), the molecular markers of TGS were observed to be sustained (Figure 8). This indicates the possibility of a long term sustainability of TGS, even when shRNA levels have declined, although the persistence of TGS in the absolute absence of shRNA could not be determined because of the extensive cell death on day 7. In this study we observed that TGS could result in the reduction of *c-Myc* for up to 6 days after single transfection.

By ChIP assay and bisulfite PCR/DNA sequencing, we demonstrated that the shRNA induces both histone and DNA methylation in HepG2 cells, which is accompanied by reduced *c-Myc* promoter acetylation (Figure 8). This was also confirmed by RT-PCR, since the test shRNA failed to decrease *c-Myc* transcript levels significantly in cells pretreated with both AZA and TSA (Additional file 5: Figure S12). In our earlier report, we were successfully able to induce TGS in glioma cell line U87 and this was shown to be by DNA methylation [34]. The current study is based on HCC cells and involves both heterochromatization and DNA methylation. It is possible that the variation in HDAC involvement is related to the cell type. As the primary message in the paper is related to the internalization of cargo via the ASGPRs, we have not explored the subtle differences in the mechanism of *c-Myc* TGS in this study.

Here we have demonstrated two levels of specificity by combining a liver cell specific delivery system with a hepatocarcinoma specific promoter/enhancer system. The effector arm of the system is the shRNA inducing TGS of *c-Myc*. With this we have been able to demonstrate silencing of the *c-Myc*, specifically in transformed liver cells, leading to extensive cell death. It is expected that combined cell delivery/transformation specific gene expression system, would be a prototype for therapeutic gene delivery in transformed cells. The shRNA inducing TGS of *c-Myc,* would also serve as an effective mechanism for inducing cell death in the targeted cells.

## Conclusions

The dual specificity resulting from Sendai F-virosomal delivery and tumour specific activation offers a novel mode of targeting HCC at two levels, first by targeted liver cell specific delivery and secondly by promoter/enhancer driven expression only in transformed hepatocarcinoma cells. Such approaches might also be utilized for other therapeutic modalities that are based on specific gene transcription e.g. Gene dependent enzyme pro-drug therapy (GDEPT). shRNA induced suppression of *c-Myc* expression by TGS is a possible gene therapy modality that could be utilized in such a delivery system. In the long run, such a targeting system may also be considered for introducing specific genes for expression in the embryonic liver or putting a check on recalcitrant cancer cells with deregulated *c-Myc*.

## Electronic supplementary material

Additional file 1: Figure S1: Molecular characterization of Chang Liver cell line. (PDF 36 KB)

Additional file 2: Figure S2: Clones of various AFP promoter/enhancer driven luciferase constructs. (A) AFPPr + 25 – luc clone was confirmed by restriction digestion with MluI and NheI restriction enzymes. (B) AFPEn-Pr + 25 - luc was digested with KpnI and MluI restriction endonulceases and (C) NFκBEn-Pr + 25 - luc with KpnI and NheI. **Figure S3.** Sequence of *c-Myc* P2 promoter with siRNA target site and CpG islands. *GAA* *CG* *GAGGGAGGGAT* *CGCG* *CT* is the siRNA target site for P2 promoter of *c-myc* and contain CpG sites 8, 9 and 10. CpG sites are highlighted in red. TATAAAAG represents the TATA box. **Figure S4.** AFP promoter – *c-myc* shRNA (AFPPr + 2 – myc) clone. (A) Schematic representation for cloning of AFPPr + 2 – myc construct. (B) Annealed *c-myc* sense and antisense oligos. (C) AFPPr + 2 – myc clone confirmation by digestion with EcoRI and HindIII restriction enzymes. **Figure S5.** NFκB – *c-myc* shRNA (NFκBEn–Pr + 2 – myc) clone. (A) Cloning strategy followed for the generation of this construct. (B) NFκB conjugated *c-myc* shRNA was confirmed by EcoRI and HindIII digestion. **Figure S6.** AFP enhancer – AFP promoter – *c-myc* shRNA (AFPEn–Pr + 2 – myc) clone. (A) Cloning strategy followed for the generation of this construct. (B) AFP enhancer and promoter conjugated *c-myc* shRNA was confirmed by EcoRI and HindIII digestion. (PDF 407 KB)

Additional file 3: Table S1: Sequence of the primers used in the study. (PDF 49 KB)

Additional file 4: Figure S7: Sequences of AFP promoter, enhancer and NFκB response element used in the study. (A) AFP Promoter sequence from – 230 to +25 bp. (B) AFP Enhancer. (C) Sequence of NFκB responsive element (4 x 10 copies). **Figure S8.** Cell survival of Huh7 cells, by MTT assay, following TGS of *c-Myc*. Following *c-Myc* suppression, Huh7 cells showed decreased cell survival but to a lesser degree when compared to that of HepG2. **Figure S9.** Cell survival of Huh7 cells, by Trypan Blue based cell counting, post *c-Myc* shRNA treatment. On the 6th day post transfection of all AFP promoter/enhancer driven *c-Myc* shRNA constructs, the decrease in cell survival of Huh7 corroborated with the MTT assay (p < 0.05). **Figure S10.** Evaluation of apoptosis in Huh7 cells by flow cytometry. Percentage of apoptotic cells, after *c-Myc* suppression *via* TGS, was dependent upon the strength of each construct driving shRNA expression. **Figure S11.** Evaluation of Interferon response, in HepG2 cells, at various time point post F-virosomal delivery of c-Myc shRNA constructs. No significant increase in the levels of OAS1 was observed in 24, 48, 72 and 96 hours post virosomal delivery of the entrapped shRNA plasmids (p > 0.05 at all points). (PDF 371 KB)

Additional file 5: Figure S12: Evaluation of *c-Myc* levels in HepG2 cells, pretreated with AZA/TSA or both in combination, followed by *c-Myc* shRNA transfection. HepG2 cells pretreated with TSA/AZA or both simultaneously were transfected with AFPEn – Pr + 2 – myc and AFPEn–Pr + 2 – myc Scr. On the 6^th^ day, real time PCR was done to evaluate the *c-Myc* transcript levels. Significant decrease in the *c-Myc* levels were observed in both AZA + AFPEn – Pr + 2 – myc and TSA + AFPEn – Pr + 2 – myc treated HepG2 cells (p < 0.05 for both). Combined treatment of both AZA + TSA along with AFPEn – Pr + 2 – myc showed no decrease in *c-Myc* levels (p > 0.05). This confirmed that shRNA induces recruitment of both HDACs and DNMTs which play their part in *c-Myc* down-regulation. **Figure S13.** Determination of shRNA expression in HepG2 cells at various time intervals by RT-PCR. c-Myc shRNA expression level was determined at various time points post transfection of c-Myc shRNA constructs. The expression of shRNA, by AFPEn – Pr + 2 – myc, was found to be maximum in 48 hours. The expression decreased significantly with time and was the lowest on day 6 (18% of the maximum on day 2; p < 0.05). shRNA, against luciferase mRNA, driven by CMV promoter (CMVPr – luc shRNA) was utilized as a control on day 1. (PDF 125 KB)

## Abbreviations

HCC: Hepatocellular carcinoma
AFP: Alpha-fetoprotein
F-protein: Fusion protein
ASGPRs: Asialoglycoprotein receptors
HN: Hemagglutinin neuraminidase
hUGT1A1: Human uridinediphosphoglucuronate glucuronosyltransferase-1A1
CEA: Carcinogenic embryonic antigen
PSA: Prostate specific antigen
siRNA: Small interfering RNA
PTGS: Post-transcriptional gene silencing
dsRNA: Double stranded RNA
TGS: Transcriptional gene silencing
H3K9Me2: Histone three lysine nine dimethylated
H3K27Me3: Histone three lysine twenty seven trimethylated
shRNA: Short hairpin RNA
NFκB: Nuclear factor kappa beta
Pol III: Polymerase III
Pol II: Polymerase II
ATCC: American type cell culture
NCCS: National center for cell science
DMEM: Dulbecco’s modified eagle’s medium
bp: Base pairs
SV40: Simian virus 40
mM: Millimolar
EDTA: Ethylenediaminetetraacetic acid
TSS: Transcription start site
RNAi: RNA interference
Scr: Scrambled
CMV: Cytomegalovirus
RT-PCR: Real-time PCR
GAPDH: Glyceraldehyde 3-phosphate dehydrogenase
REST: Relative expression software tool
MTT: 3-(4,5-dimethylthiazol-2-yl)-2,5-diphenyltetrazolium bromide
PI: Propidium Iodide
SDS: Sodium dodecyl sulphate
R18: Rhodamine beta chloride
PBS: Phosphate buffered saline
rpm: Revolutions per minute
DTT: dithiothreitol
ChIP: Chromatin immunoprecipitation assay
HDAC: Histone deacetylase
DNMT: DNA methyl transferase
TSA: Trichostatin A
AZA: 5-aza-2deoxycytidine
HC: Heat control
IFN: Interferon
OAS1: Oligo adenylate synthetase 1
hTERT: Human telomerase reverse transcriptase
GDEPT: Gene dependent enzyme pro-drug therapy.
