# Supplementary material for: Combination of hepatocyte specific delivery and transformation dependent expression of shRNA inducing transcriptional gene silencing of c-Myc promoter in hepatocellular carcinoma cells
Source: BMC Cancer. 2014 Aug 10;14:582. doi: 10.1186/1471-2407-14-582 (PMC4153911; doi:10.1186/1471-2407-14-582)
Supplement: Supplementary file 1 — Additional file 1: Figure S1: Molecular characterization of Chang Liver cell line. (PDF 36 KB) [file 12885_2014_4798_MOESM1_ESM.pdf]

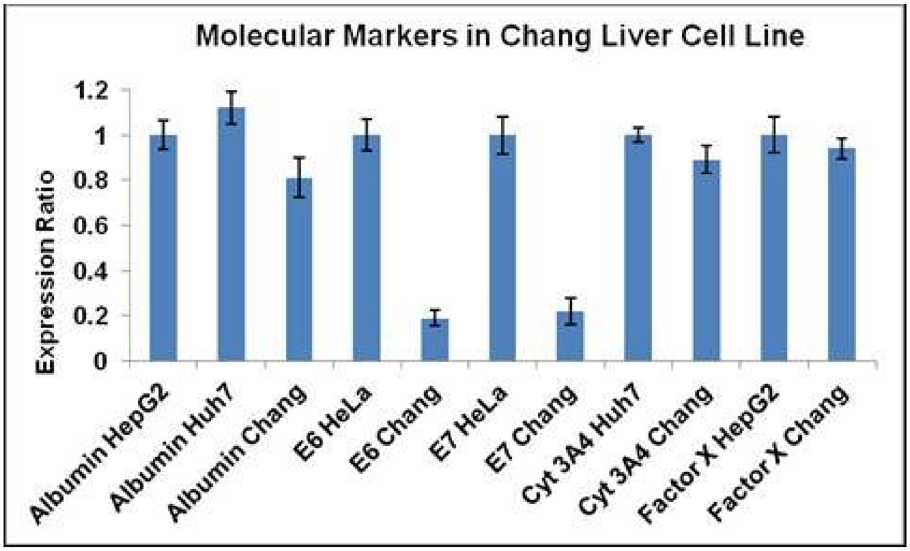

Additional file 1: Figure S1. Molecular characterization of Chang Liver cell line. Before experimentation on Chang Liver, for its authentication, we checked the expression level of various liver specific factors by real-time PCR. Significant expression of Albumin, Cytochrome C3A4 and Factor X was observed in Chang Liver cells when compared to either HepG2/Huh7 cells. However, significant difference in the level of E6/E7 HPV18 oncoproteins ($p = 0.0041$ and $p = 0.0027$ for E6 and E7) was observed in Chang Liver cells when compared with that of HeLa cells. We also confirmed the presence of liver specific surface asialoglycoprotein receptors (ASGPR) on Chang Liver by Sendai virosomal fusion studies (Figure 6A). Additionally, Park and coworkers (2000) have shown fusion of galactosylated chitosan-dextran-DNA with Chang Liver and HepG2 through ASGPR [1]. Lastly, since we observed no luciferase activity through AFP promoter/enhancer mediated constructs in Chang Liver cell line, this cell line served as an untransformed control model for our experiments.

References

1. Pathak A, Vyas SP, Gupta KC: **Nano-vectors for efficient liver specific gene transfer**. *Int J Nanomedicine* 2008, **3**:31–49.
