# Supplementary material for: Combination of hepatocyte specific delivery and transformation dependent expression of shRNA inducing transcriptional gene silencing of c-Myc promoter in hepatocellular carcinoma cells
Source: BMC Cancer. 2014 Aug 10;14:582. doi: 10.1186/1471-2407-14-582 (PMC4153911; doi:10.1186/1471-2407-14-582)
Supplement: Supplementary file 2 — Additional file 2: Figure S2: Clones of various AFP promoter/enhancer driven luciferase constructs. (A) AFPPr + 25 – luc clone was confirmed by restriction digestion with MluI and NheI restriction enzymes. (B) AFPEn-Pr + 25 - luc was digested with KpnI and MluI restriction endonulceases and (C) NFκBEn-Pr + 25 - luc with KpnI and NheI. Figure S3. Sequence of c-Myc P2 promoter with siRNA target site and CpG islands. GAA CG GAGGGAGGGAT CGCG CT is the siRNA target site for P2 promoter of c-myc and contain CpG sites 8, 9 and 10. CpG sites are highlighted in red. TATAAAAG represents the TATA box. Figure S4. AFP promoter – c-myc shRNA (AFPPr + 2 – myc) clone. (A) Schematic representation for cloning of AFPPr + 2 – myc construct. (B) Annealed c-myc sense and antisense oligos. (C) AFPPr + 2 – myc clone confirmation by digestion with EcoRI and HindIII restriction enzymes. Figure S5. NFκB – c-myc shRNA (NFκBEn–Pr + 2 – myc) clone. (A) Cloning strategy followed for the generation of this construct. (B) NFκB conjugated c-myc shRNA was confirmed by EcoRI and HindIII digestion. Figure S6. AFP enhancer – AFP promoter – c-myc shRNA (AFPEn–Pr + 2 – myc) clone. (A) Cloning strategy followed for the generation of this construct. (B) AFP enhancer and promoter conjugated c-myc shRNA was confirmed by EcoRI and HindIII digestion. (PDF 407 KB) [file 12885_2014_4798_MOESM2_ESM.pdf]

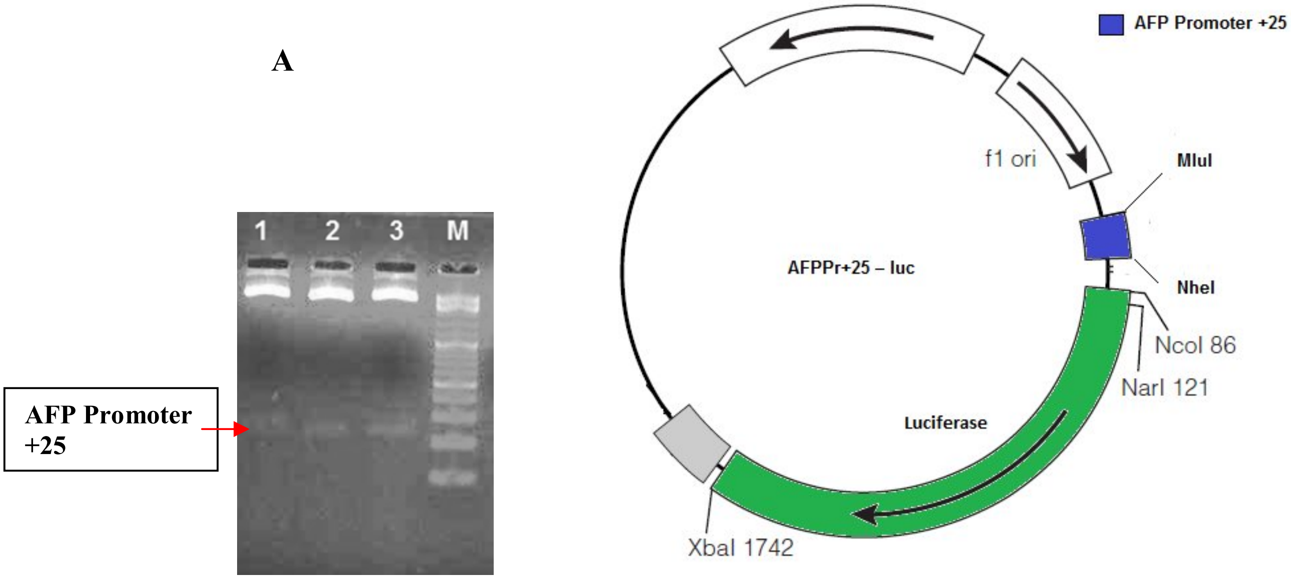
A
1
2
3
M
AFP Promoter +25
AFP Promoter +25
f1 ori
MluI
NheI
AFPPr+25 – luc
NcoI 86
NarI 121
Luciferase
XbaI 1742

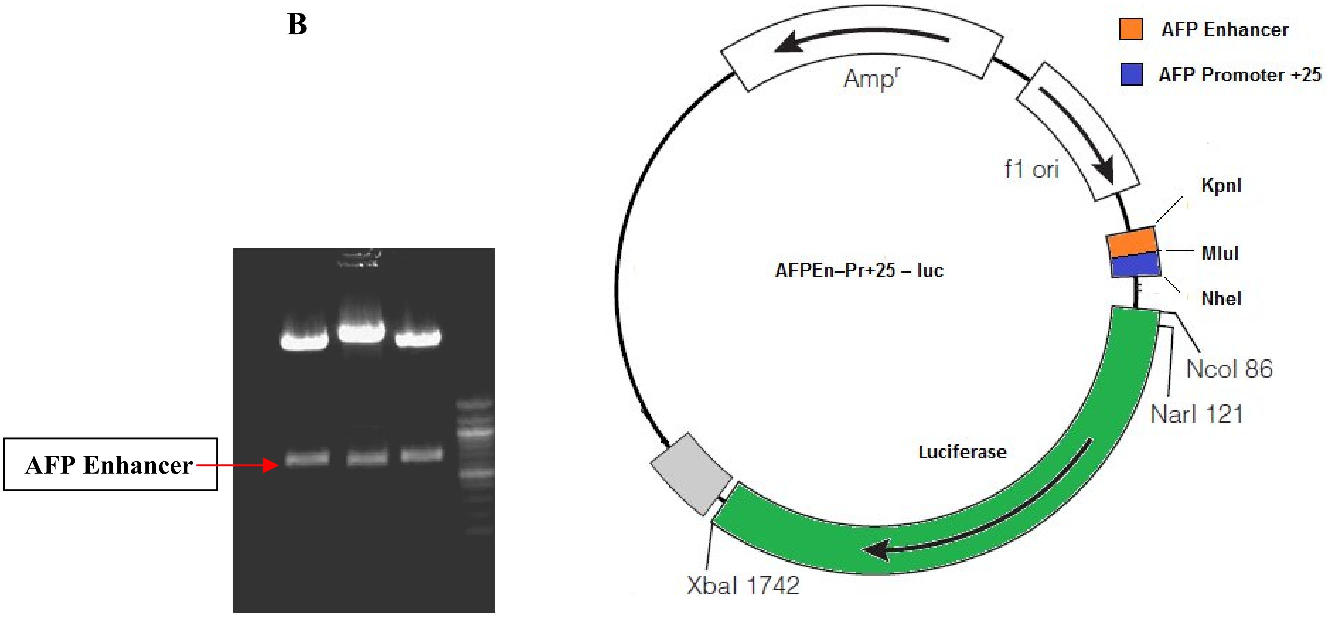
B
AFP Enhancer
AFP Enhancer
AFP Promoter +25
Amp^r
f1 ori
KpnI
MluI
NheI
AFPEn–Pr+25 – luc
NcoI 86
NarI 121
Luciferase
XbaI 1742

C

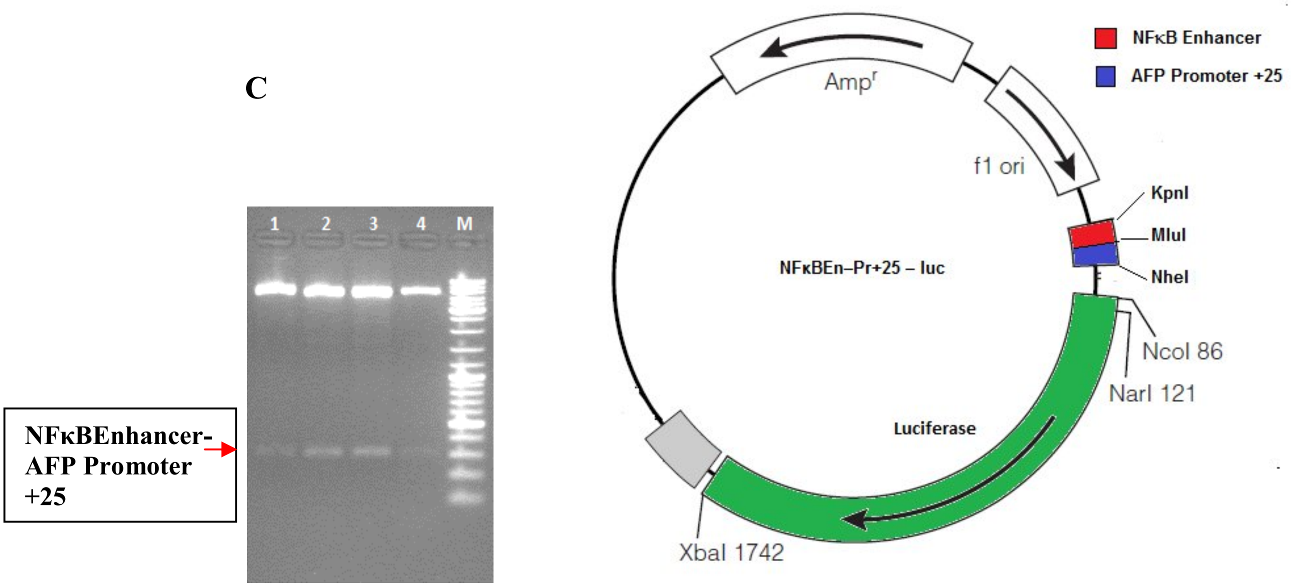

Additional file 2: Figure S2. Clones of various AFP promoter/enhancer driven luciferase constructs. (A) AFPPr+25 – luc clone was confirmed by restriction digestion with MluI and NheI restriction enzymes. **(B)** AFPEn-Pr+25 - luc was digested with KpnI and MluI restriction endonulceases and **(C)** NFκBEn-Pr+25 - luc with KpnI and NheI.

CpG1

CCGCCACCGCCGGGCCCCGGCCGTCCCTGGCTCCCCTCCTGCCTCGAGAAGGGCAGGGCTTCTCAGAGGCTTGGC

GGGAAAAAGAACGGAGGGAGGGATCGCGCTGAGTATAAAAGCCGGTTTTCGGGGCTTTATCTAACTCGCTGTAGT

AATTCCAGCGAGAGGCAGAGGGAGCGAGCGGGCGGCCGGCTAGGGTGGAAGAGCCGGGCGA

CpG20

Additional file 2: Figure S3. Sequence of *c-Myc* P2 promoter with siRNA target site and CpG islands. **GAACGGAGGGAGGGATCGCGCT** is the siRNA target site for P2 promoter of *c-myc* and contain CpG sites 8, 9 and 10. CpG sites are highlighted in red. TATAAAAG represents the TATA box.

A

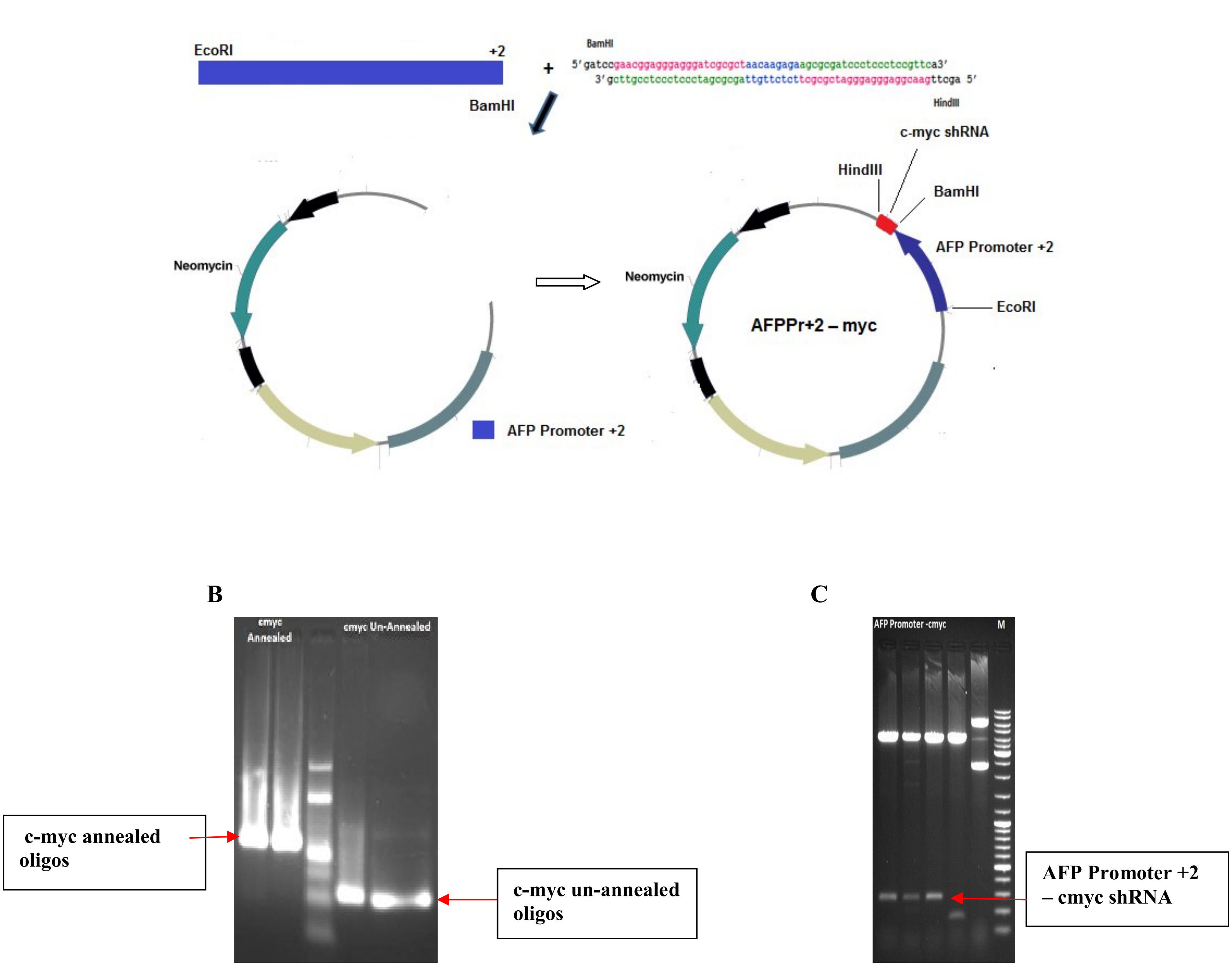

Additional file 2: Figure S4. AFP promoter – *c-myc* shRNA (AFPPr+2 – myc) clone. (A) Schematic representation for cloning of AFPPr+2 – myc construct. **(B)** Annealed *c-myc* sense and antisense oligos. **(C)** AFPPr+2 – myc clone confirmation by digestion with EcoRI and HindIII restriction enzymes.

A

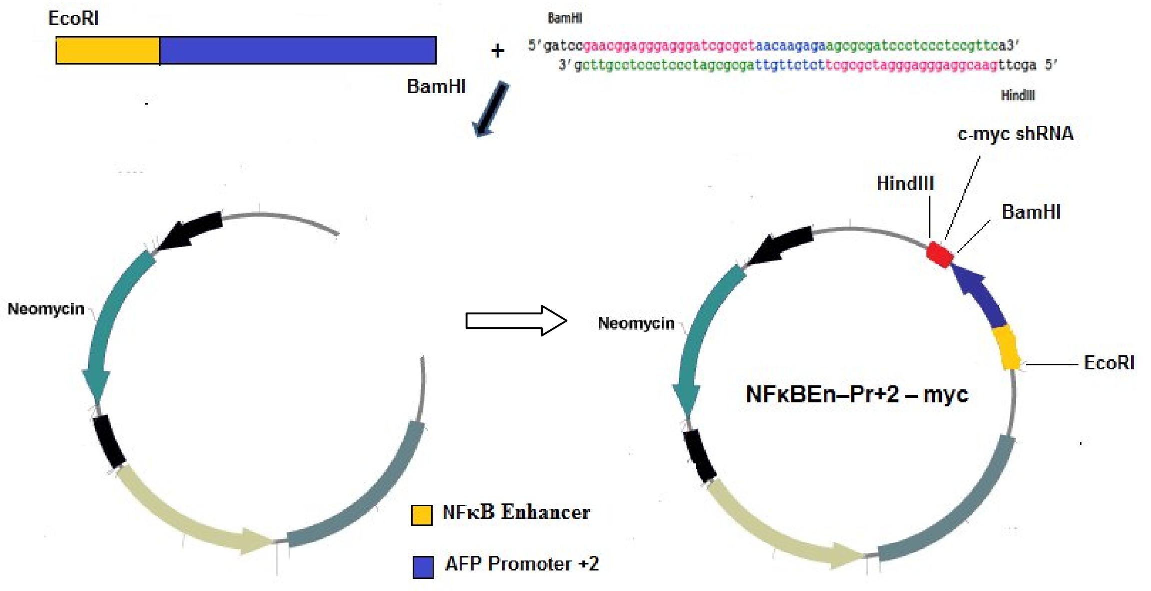

B

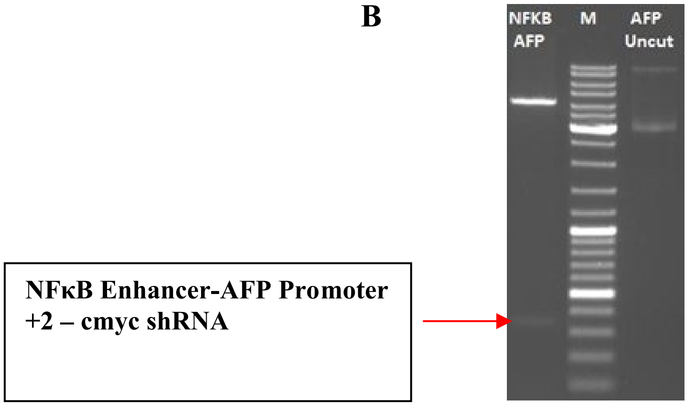

Additional file 2: Figure S5. NFκB – *c-myc* shRNA (NFκBEn–Pr+2 – myc) clone. (A) Cloning strategy followed for the generation of this construct. **(B)** NFκB conjugated *c-myc* shRNA was confirmed by EcoRI and HindIII digestion.

A

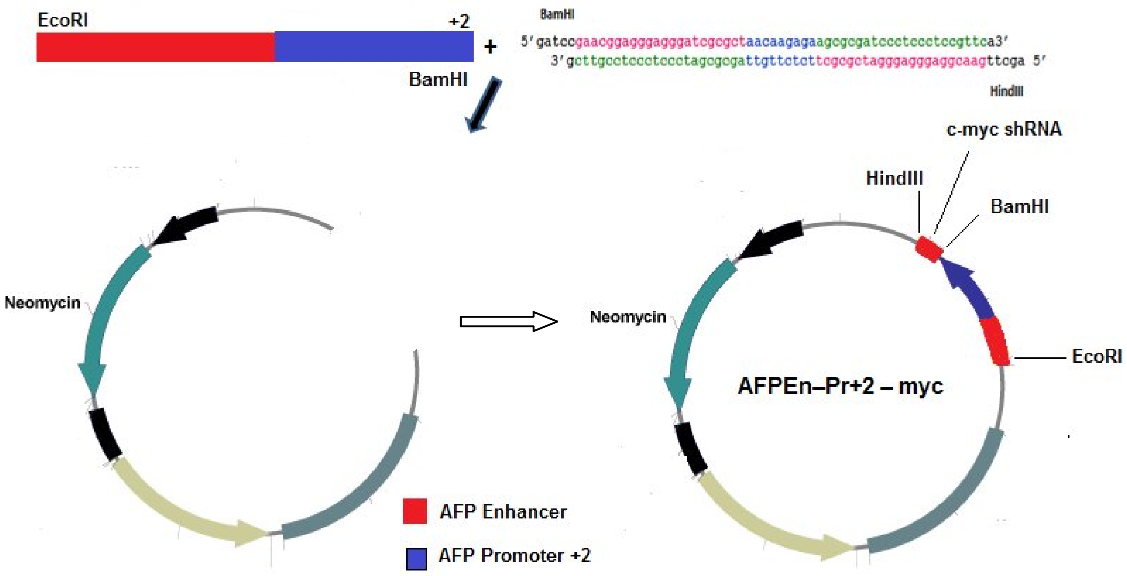

B

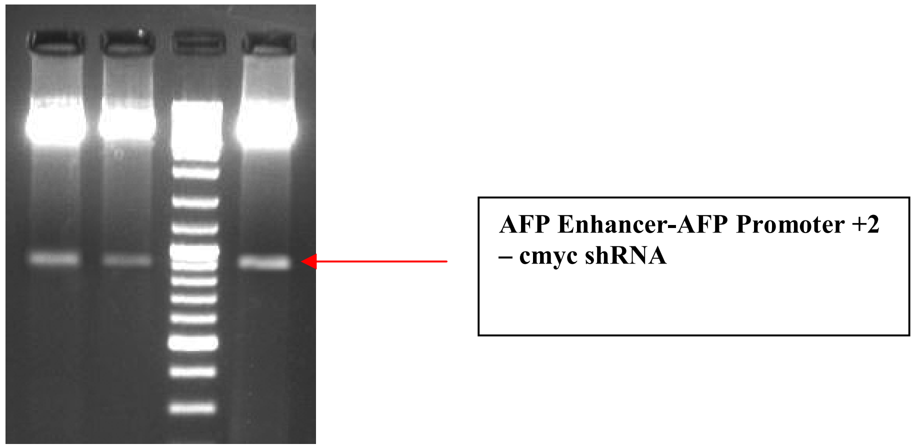

Additional file 2: Figure S6. AFP enhancer – AFP promoter – *c-myc* shRNA (AFPEn–Pr+2 – myc) clone. (A) Cloning strategy followed for the generation of this construct. **(B)** AFP enhancer and promoter conjugated *c-myc* shRNA was confirmed by EcoRI and HindIII digestion.

(All clones were verified by DNA sequencing from professional agencies)
