# Supplementary material for: Combination of hepatocyte specific delivery and transformation dependent expression of shRNA inducing transcriptional gene silencing of c-Myc promoter in hepatocellular carcinoma cells
Source: BMC Cancer. 2014 Aug 10;14:582. doi: 10.1186/1471-2407-14-582 (PMC4153911; doi:10.1186/1471-2407-14-582)
Supplement: Supplementary file 3 — Additional file 3: Table S1: Sequence of the primers used in the study. (PDF 49 KB) [file 12885_2014_4798_MOESM3_ESM.pdf]

Additional file 3: Table S1. Sequence of the primers used in the study.

Name	Sequence 5' to 3'
**NF-κB Response Element (Enhancer)**	**GGGAATTTCCGGGAATTTCCGGGAATTTCCGGGAATTTCC**
***c-myc* (F) (real time PCR**	**GCTGCTATGGGCAAAGTTTC**
***c-myc* (R) (real time PCR)**	**AGGGCTTCTCAGAGGCTTG**
***c-myc* ChIP (F) (real time PCR)**	**CCCACCGGCCCTTTATAATGC**
***c-myc* ChIP (R) (real time PCR)**	**GCTCGGGTGTTGTAAGTTCC**
***c-myc* outer (F) (Bisulphite nested PCR)**	**TTTGAGAGGGAGTAAAAG**
***c-myc* outer (R) (Bisulphite nested PCR)**	**TTCCAATACAAAATACCC**
***c-myc* inner (F) (Bisulphite nested PCR)**	**CGCCAGGGTTTTCCCAGTCACGACGTGGGAAAAGAAAAAAG**
***c-myc* inner (R) (Bisulphite nestedPCR)**	**AGCGGATAACAATTTCACACAGGAAACCAAAAACRAAACCCC**
**Chr16 (F) ChIP**	**GTCTCTTTCTTGTTTTTAAGCTGGG**

**Chr16 (R) ChIP**	**TGAGCTCATTGAGACATTTGG**
**GAPDH (F) (real time PCR)**	**CCAAGGTCATCCATGACAACTTTGGT**
**GAPDH (R) (real time PCR)**	**TGTTGAAGTCAGAGGAGACCACCTG**
**β-Actin (F) (real time PCR)**	**TCATGAAGTGTGACGTTGACATCCGT**
**β-Actin (R) (real time PCR)**	**CCTAGAAGCATTTGCGGTGCACGATG**
**AFP Enhancer (F)**	**TGACCATAAGACAATTAGAT**
**AFP Enhancer (R)**	**GGATAAAGCTGAGTGGTATGAA**
**NFκB-AFP+2 (F)**	**CGGAATTCATTCACGCGTGGGAATTT**
**NFκB-AFP+2 (R)**	**AGGGATCCATGGAAAATCATGCTGAAATTCTTT**
**AFP Promoter +2 (F)**	**GGTACCGAATATTTGTTATATTTGCA**
**AFP Promoter +2 (R)**	**AGGGATCCATGGAAAATCATGCTGAAATTCTTT**
**AFPEn-AFPPr +2 (F)**	**GAATTCTTCACCATAACACAATTAGAT**
**AFPEn-AFPPr +2 (R)**	**AGGGATCCATGGAAAATCATGCTGAAATTCTTT**
**M13 (F) (sequencing)**	**CGCCAGGGTTTTCCCAGTCACGAC**
**M13 (R)**	**AGCGGATAACAATTTCACACAGGA**

(sequencing)	
OAS1 (F) (real time PCR)	TTCTCCACCTGCTTCACAGA
OAS1 (R) (real time PCR)	GAGCTCCAGGGCATACTGAG
18S (F) (real time PCR)	GTAACCCGTTGAACCCCATT
18S (R) (real time PCR)	CCATCCAATCGGTAGTAGCG
Sequencing Primer PGl3 Basic (F)	CTAGCAAAATAGGCTGTCCC
Sequencing Primer PGl3 Basic (R)	CTTTATGTTTTTGGCGTCTTCCA
hTERT (F)	GGAGCAAGTTGCAAAGCATTG
hTERT (R)	TCCCACGACGTAGTCCATGTT
Cyclin D3 (F)	ATGCTCCGAGGCTAAGACTC
Cyclin D3 (R)	GAGAGAGTGTCCTTGGTCCC

**Albumin (F)**	**TATGCCCCGGAACTCCTTTT**
**Albumin (R)**	**TGGCACACTTGAGTCTCTGT**
**HPV-18 E6 (F)**	**CATGCTGCATGCCATAAATG**
**HPV-18 E6 (R)**	**TGTGTTTCTCTGCGTCGTTG**
**HPV-18 E7 (F)**	**ACCTAAGGCAACATTGCAAG**
**HPV-18 E7 (R)**	**ACAAAGGACAGGGTGTTCAG**
**Cyt 3A4 (F)**	**AGCCTCACTGAATCACTGCT**
**Cyt 3A4 (R)**	**GGTTTCCATGGCCAAGTCTG**
**Factor X (F)**	**TGGAACCATTCTGAGCGAGT**

**Factor X (R)**	**AACCGGTTGTGCTTGATGAC**
**c-Myc siRNA forward primer**	**GAACGGAGGGAGGGAUCGCGC**
