# Supplementary material for: Combination of hepatocyte specific delivery and transformation dependent expression of shRNA inducing transcriptional gene silencing of c-Myc promoter in hepatocellular carcinoma cells
Source: BMC Cancer. 2014 Aug 10;14:582. doi: 10.1186/1471-2407-14-582 (PMC4153911; doi:10.1186/1471-2407-14-582)
Supplement: Supplementary file 4 — Additional file 4: Figure S7: Sequences of AFP promoter, enhancer and NFκB response element used in the study. (A) AFP Promoter sequence from – 230 to +25 bp. (B) AFP Enhancer. (C) Sequence of NFκB responsive element (4 x 10 copies). Figure S8. Cell survival of Huh7 cells, by MTT assay, following TGS of c-Myc. Following c-Myc suppression, Huh7 cells showed decreased cell survival but to a lesser degree when compared to that of HepG2. Figure S9. Cell survival of Huh7 cells, by Trypan Blue based cell counting, post c-Myc shRNA treatment. On the 6th day post transfection of all AFP promoter/enhancer driven c-Myc shRNA constructs, the decrease in cell survival of Huh7 corroborated with the MTT assay (p < 0.05). Figure S10. Evaluation of apoptosis in Huh7 cells by flow cytometry. Percentage of apoptotic cells, after c-Myc suppression via TGS, was dependent upon the strength of each construct driving shRNA expression. Figure S11. Evaluation of Interferon response, in HepG2 cells, at various time point post F-virosomal delivery of c-Myc shRNA constructs. No significant increase in the levels of OAS1 was observed in 24, 48, 72 and 96 hours post virosomal delivery of the entrapped shRNA plasmids (p > 0.05 at all points). (PDF 371 KB) [file 12885_2014_4798_MOESM4_ESM.pdf]

A

AFP Promoter +25

GGTACCGAATATTTGTTATATTTGCAAAATAAAATAAGTTTGCAAGTTTTTTTTTTCTGCCCCAAAGAGCTCTGTGT
CCTTGAACATAAAATACAAATAACCGCTATGCTGTTAATTATTGGCAAATGTCCCATTTTCAACCTAAGGAAATACC
ATAAAGTAACAGATATACCAACAAAAGGTTACTAGTTAACAGGCATTGCCTGAAAAGAGTATAAAAGAATTTCAGCA
TGATTTTCCATATTGTGCTTCCAC

B

AFP Enhancer 700 bp

ATTAGTTTTGAATCTTTCTAATACCAAAGTTCAGTTTACTGTTCCATGTTGCTTCTGAGTGGCTTCACAGACTTATG
AAAAAGTAAACGGAATCAGAATTACATCAATGCAAAAGCATTGCTGTGAACTCTGTACTTAGGACTAAACTTTGAGC
AATAACACATATAGATTGAGGATTGTTTGCTGTTAGTATACAAACTCTGGTTCAAAGCTCCTCTTTATTGCTTGTCT
TGGAAAATTTGCTGTTCTTCATGGTTTCTCTTTTCACTGCTATCTATTTTTCTCAACCACTCACATGGCTACAATAA
CTGTCTGCAAGCTTATGATTCCCAAATATCTATCTCTAGCCTCAATCTTGTTCCAGAAGATAAAAAGTAGTATTCAA
ATGCACATCAACGTCTCCACTTGGAGGGCTTAAAGACGTTTCAACATACAAACCGGGGAGTTTTGCCTGGAATGTTT
CCTAAAATGTGTCCTGTAGCACATAGGGTCCTCTTGTTCCTTAAAATCTAATTACTTTTAGCCCAGTGCTCATCCCA
CCTATGGGGAGATGAGAGTGAAAAGGGAGCCTGATTAATAATTACACTAAGTCAATAGGCATAGAGCCAGGACTGTT
TGGGTAAACTGGTCACTTTATCTTAAACTAAATATATCCAAAACTGAACATGTACTTAGTTACTAAGTCTTTGACTT
TATCTCA

C

NFκB Response Element

GGGAATTTCCGGGAATTTCCGGGAATTTCCGGGAATTTCC

Additional file 4: Figure S7. Sequences of AFP promoter, enhancer and NFκB response element used in the study. (A) AFP Promoter sequence from – 230 to +25 bp. **(B)** AFP Enhancer. **(C)** Sequence of NFκB responsive element (4 x 10 copies).

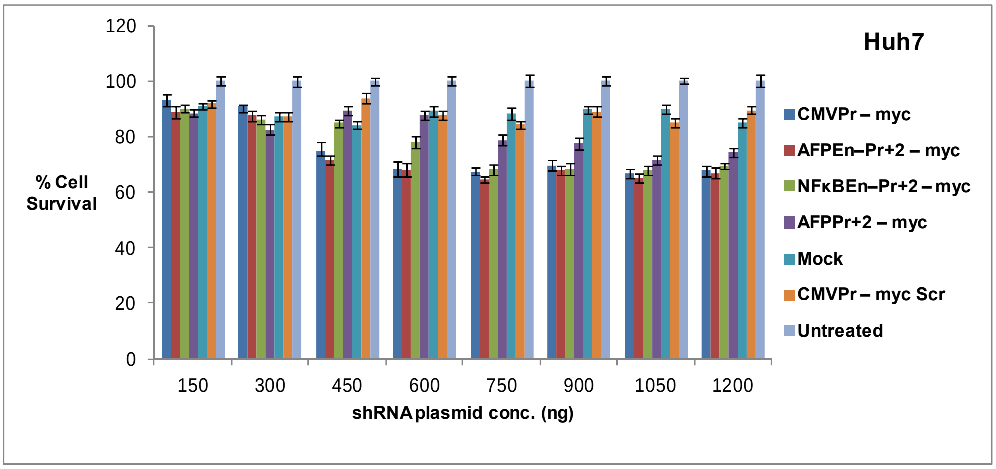

Additional file 4: Figure S8. **Cell survival of Huh7 cells, by MTT assay, following TGS of *c-Myc***. Following *c-Myc* suppression, Huh7 cells showed decreased cell survival but to a lesser degree when compared to that of HepG2.

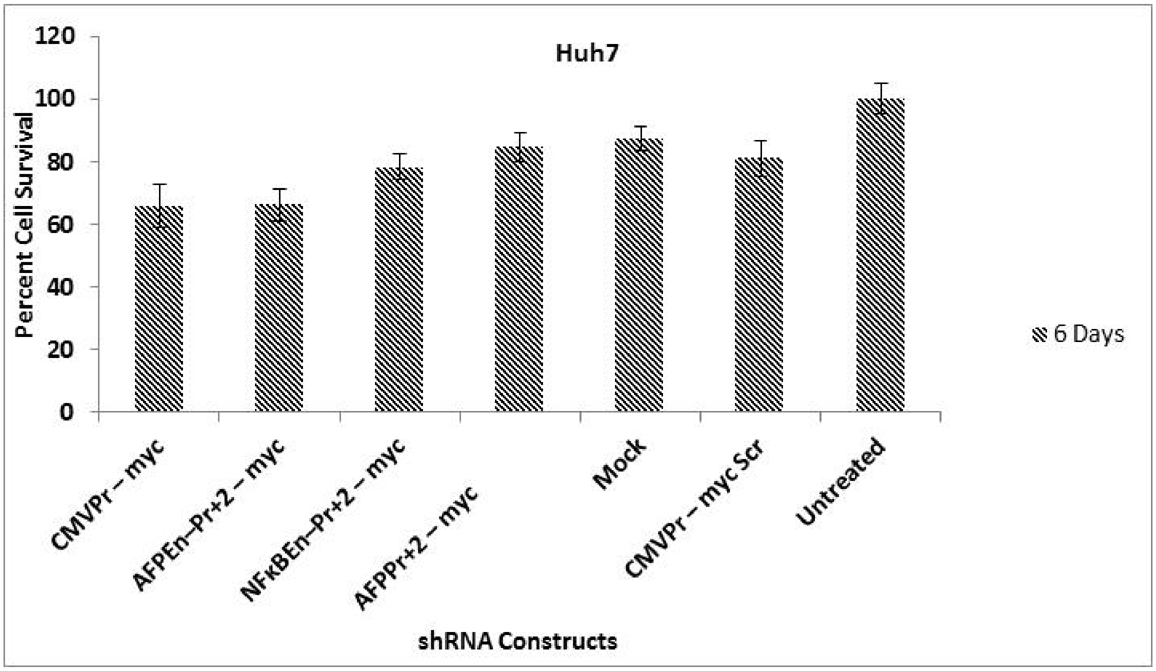

Additional file 4: Figure S9. **Cell survival of Huh7 cells, by Trypan Blue based cell counting, post *c-Myc* shRNA treatment**. On the 6th day post transfection of all AFP promoter/enhancer driven *c-Myc* shRNA constructs, the decrease in cell survival of Huh7 corroborated with the MTT assay ($p < 0.05$).

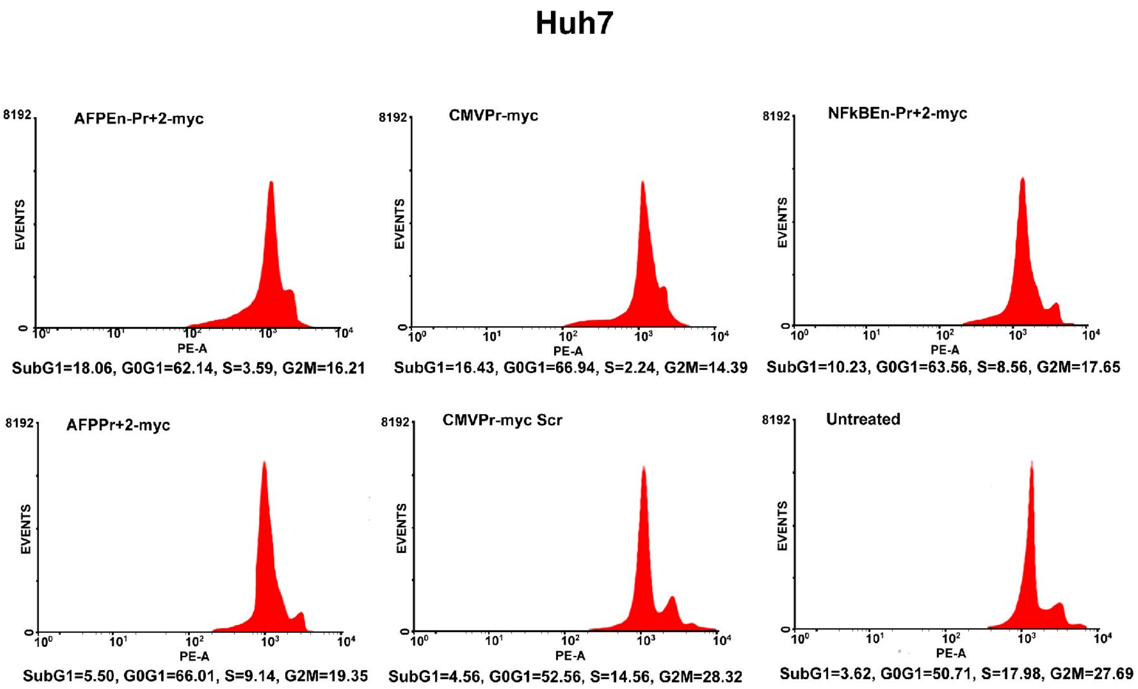

Additional file 4: Figure S10. **Evaluation of apoptosis in Huh7 cells by flow cytometry**. Percentage of apoptotic cells, after *c-Myc* suppression *via* TGS, was dependent upon the strength of each construct driving shRNA expression.

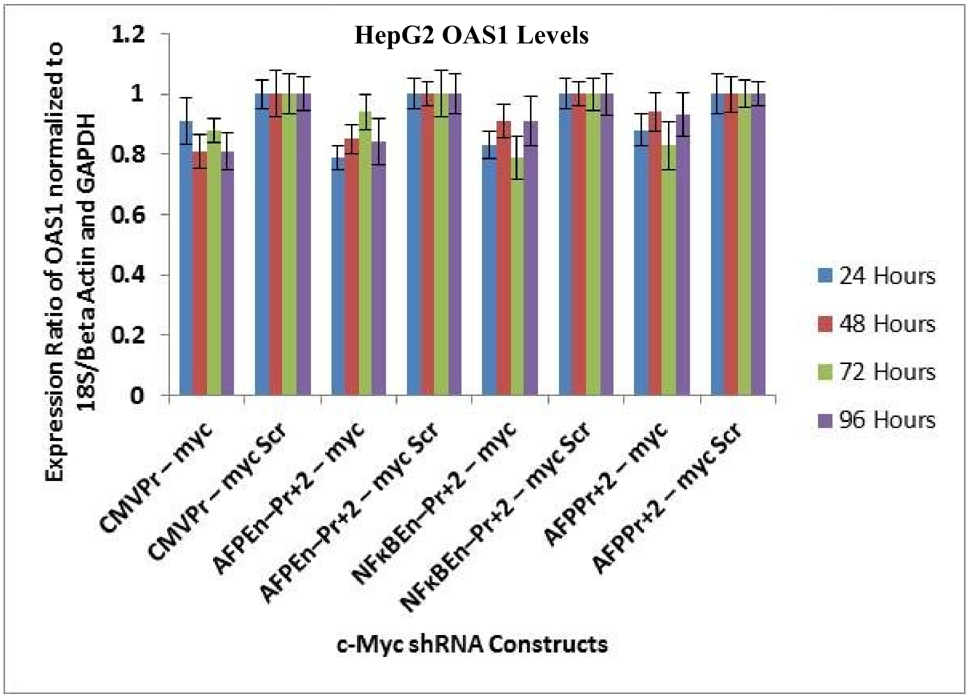

Additional file 4: Figure S11. **Evaluation of Interferon response, in HepG2 cells, at various time point post F-virosomal delivery of c-Myc shRNA constructs.** No significant increase in the levels of OAS1 was observed in 24, 48, 72 and 96 hours post virosomal delivery of the entrapped shRNA plasmids ($p > 0.05$ at all points).
