# Supplementary material for: Combination of hepatocyte specific delivery and transformation dependent expression of shRNA inducing transcriptional gene silencing of c-Myc promoter in hepatocellular carcinoma cells
Source: BMC Cancer. 2014 Aug 10;14:582. doi: 10.1186/1471-2407-14-582 (PMC4153911; doi:10.1186/1471-2407-14-582)
Supplement: Supplementary file 5 — Additional file 5: Figure S12: Evaluation of c-Myc levels in HepG2 cells, pretreated with AZA/TSA or both in combination, followed by c-Myc shRNA transfection. HepG2 cells pretreated with TSA/AZA or both simultaneously were transfected with AFPEn – Pr + 2 – myc and AFPEn–Pr + 2 – myc Scr. On the 6th day, real time PCR was done to evaluate the c-Myc transcript levels. Significant decrease in the c-Myc levels were observed in both AZA + AFPEn – Pr + 2 – myc and TSA + AFPEn – Pr + 2 – myc treated HepG2 cells (p < 0.05 for both). Combined treatment of both AZA + TSA along with AFPEn – Pr + 2 – myc showed no decrease in c-Myc levels (p > 0.05). This confirmed that shRNA induces recruitment of both HDACs and DNMTs which play their part in c-Myc down-regulation. Figure S13. Determination of shRNA expression in HepG2 cells at various time intervals by RT-PCR. c-Myc shRNA expression level was determined at various time points post transfection of c-Myc shRNA constructs. The expression of shRNA, by AFPEn – Pr + 2 – myc, was found to be maximum in 48 hours. The expression decreased significantly with time and was the lowest on day 6 (18% of the maximum on day 2; p < 0.05). shRNA, against luciferase mRNA, driven by CMV promoter (CMVPr – luc shRNA) was utilized as a control on day 1. (PDF 125 KB) [file 12885_2014_4798_MOESM5_ESM.pdf]

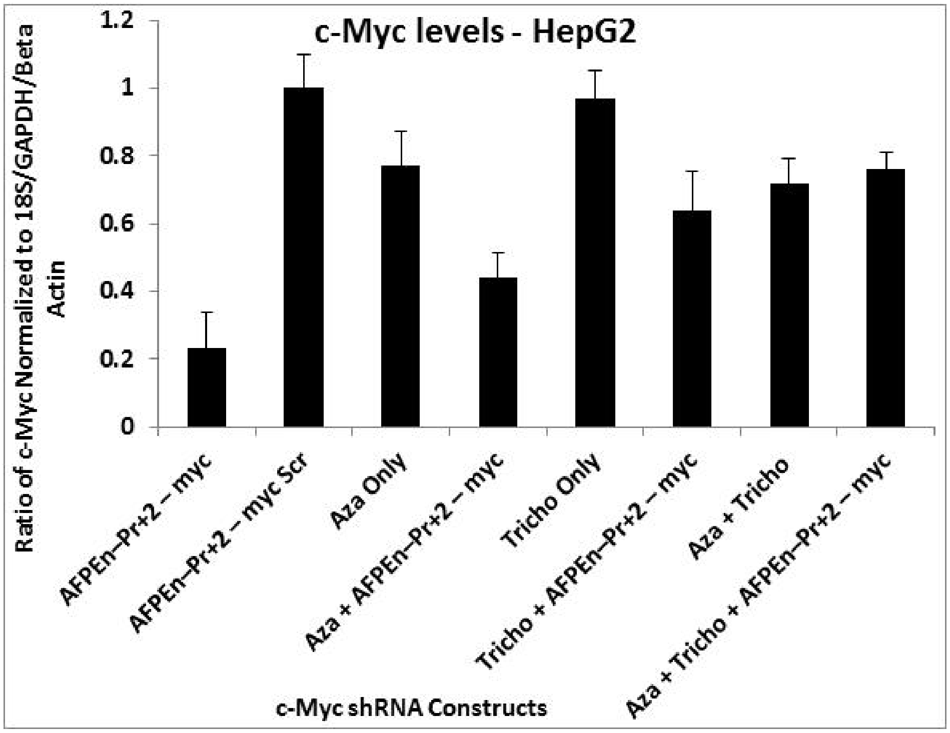

Additional file 5: Figure S12. Evaluation of *c-Myc* levels in HepG2 cells, pretreated with AZA/TSA or both in combination, followed by *c-Myc* shRNA transfection. HepG2 cells pretreated with TSA/AZA or both simultaneously were transfected with AFPEn – Pr+2 – myc and AFPEn–Pr+2 – myc Scr. On the 6th day, real time PCR was done to evaluate the *c-Myc* transcript levels. Significant decrease in the *c-Myc* levels were observed in both AZA + AFPEn – Pr+2 – myc and TSA + AFPEn – Pr+2 – myc treated HepG2 cells ($p < 0.05$ for both). Combined treatment of both AZA+TSA along with AFPEn – Pr+2 – myc showed no decrease in *c-Myc* levels ($p > 0.05$). This confirmed that shRNA induces recruitment of both HDACs and DNMTs which play their part in *c-Myc* down-regulation.

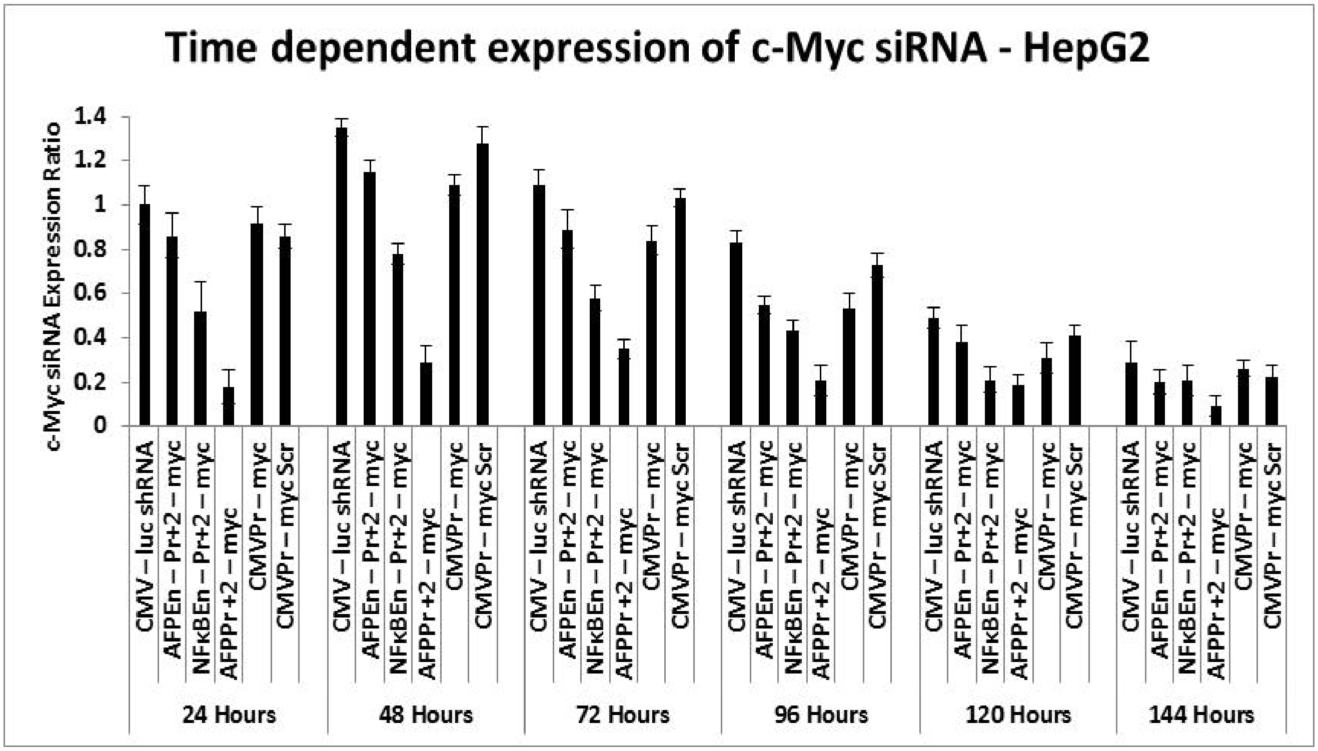

Additional file 5: Figure S13. Determination of shRNA expression in HepG2 cells at various time intervals by RT-PCR. *c-Myc* shRNA expression level was determined at various time points post transfection of *c-Myc* shRNA constructs. The expression of shRNA, by AFPEn – Pr+2 – myc, was found to be maximum in 48 hours. The expression decreased significantly with time and was the lowest on day 6 (18% of the maximum on day 2; $p < 0.05$). shRNA, against luciferase mRNA, driven by CMV promoter (CMVPr – luc shRNA) was utilized as a control on day 1.
